# Accurate Prediction of Band Structure of FeS_2_: A Hard Quest of Advanced First-Principles Approaches

**DOI:** 10.3389/fchem.2021.747972

**Published:** 2021-09-28

**Authors:** Min-Ye Zhang, Hong Jiang

**Affiliations:** Beijing National Laboratory for Molecular Sciences, College of Chemistry and Molecular Engineering, Peking University, Beijing, China

**Keywords:** iron disulfide, band structure, GW approximation, self-energy, linearized augmented plane waves, hybrid functionals

## Abstract

The pyrite and marcasite polymorphs of FeS_2_ have attracted considerable interests for their potential applications in optoelectronic devices because of their appropriate electronic and optical properties. Controversies regarding their fundamental band gaps remain in both experimental and theoretical materials research of FeS_2_. In this work, we present a systematic theoretical investigation into the electronic band structures of the two polymorphs by using many-body perturbation theory with the *GW* approximation implemented in the full-potential linearized augmented plane waves (FP-LAPW) framework. By comparing the quasi-particle (QP) band structures computed with the conventional LAPW basis and the one extended by high-energy local orbitals (HLOs), denoted as LAPW + HLOs, we find that one-shot or partially self-consistent *GW* (*G*_0_ *W*_0_ and *GW*_0_, respectively) on top of the Perdew-Burke-Ernzerhof (PBE) generalized gradient approximation with a converged LAPW + HLOs basis is able to remedy the artifact reported in the previous *GW* calculations, and leads to overall good agreement with experiment for the fundamental band gaps of the two polymorphs. Density of states calculated from *G*_0_ *W*_0_@PBE with the converged LAPW + HLOs basis agrees well with the energy distribution curves from photo-electron spectroscopy for pyrite. We have also investigated the performances of several hybrid functionals, which were previously shown to be able to predict band gaps of many insulating systems with accuracy close or comparable to *GW*. It is shown that the hybrid functionals considered in general fail badly to describe the band structures of FeS_2_ polymorphs. This work indicates that accurate prediction of electronic band structure of FeS_2_ poses a stringent test on state-of-the-art first-principles approaches, and the *G*_0_ *W*_0_ method based on semi-local approximation performs well for this difficult system if it is practiced with well-converged numerical accuracy.

## 1 Introduction

Iron disulfide FeS_2_ was studied extensively in the last century in the desire of understanding the structural and electronic properties of transition metal dichalcogenides (TMDC) featuring localized or band-like *d* electrons (Hulliger and Mooser, 1965a; Hulliger and Mooser, 1965b; Goodenough, 1972; Wilson, 1972; Li et al., 1974; Schlegel and Wachter, 1976; Folkerts et al., 1987). Since 1980s (Chatzitheodorou et al., 1986; Ennaoui et al., 1986), increasing practical interest has been drawn to pyrite FeS_2_ for its potential as a cheap and competitive candidate material for efficient solar energy conversion (Wadia et al., 2009) because of its natural abundance, non-toxicity, suitable optical gap and extraordinarily large absorption coefficient (Ferrer et al., 1990; Ennaoui et al., 1993). This has led to new solutions under various optoelectronic scenarios, including photovoltaics (Khalid et al., 2018), photo-catalysis (Tian et al., 2015; Barawi et al., 2016), solid-state photo-capacitors (Gong et al., 2013a) and photo-detectors (Wang et al., 2012; Gong et al., 2013b). However, practical application of FeS_2_-based optoelectronic devices is seriously hampered by its unexpected low efficiency due to a loss of open-circuit voltage *V*_OC_ (Cabán-Acevedo et al., 2014). A number of factors possibly responsible for the low *V*_OC_ have been suggested and examined, including the intrinsic and defect surface states (Bronold et al., 1994; Sun et al., 2011; Herbert et al., 2013; Lazić et al., 2013; Cabán-Acevedo et al., 2014; Limpinsel et al., 2014; Walter et al., 2017), bulk sulfur deficiency (Birkholz et al., 1991; Cabán-Acevedo et al., 2014; Shukla et al., 2016) and presence of the metastable marcasite phase as a small-gap impurity (Spagnoli et al., 2010; Sun et al., 2011; Schena et al., 2013).

Despite progress towards understanding the origin of the low *V*_OC_ in pyrite FeS_2_ (Rahman et al., 2020), consensus is still not reached on the fundamental band gaps of the two FeS_2_ phases. Experimentally, values varying from 0.6 to 2.6 eV have been reported for pyrite, primarily due to differences in sample preparation, measuring technique, and analytical model of spectra used in experimental studies (Ferrer et al., 1990; Ennaoui et al., 1993). Measurements of the pyrite band gap are generally carried out through optical absorption spectroscopy (Schlegel and Wachter, 1976; Kou and Seehra, 1978; Ennaoui et al., 1993), which features the neutral excitation (exciton) instead of the charged one as in the photo-electron spectroscopy (PES). Therefore, the measured excitation energies are in fact coupled to the electron-hole binding. Careful investigation by absorption spectroscopy for the marcasite phase is done only recently and gives an optical gap similar to pyrite, which essentially precludes the possibility of marcasite being the culprit for the low *V*_OC_ of FeS_2_ photovoltaics (Sánchez et al., 2016; Wu et al., 2016). Furthermore, even though PES measurements of pyrite FeS_2_ have been conducted (Ohsawa et al., 1974; van der Heide et al., 1980; Folkerts et al., 1987; Mamiya et al., 1997; Ollonqvist et al., 1997; Nesbitt et al., 2003), combined studies of direct and inverse PES (IPS) for regions near the Fermi level are rare. Reported relevant works (Folkerts et al., 1987; Mamiya et al., 1997) were done more than 20 years ago and the spectra were not resolved enough to identify a well-defined fundamental band gap.

Difficulties in characterizing band structures of FeS_2_ polymorphs are also encountered from the perspective of first-principles calculations. Within the framework of density functional theory (DFT) (Hohenberg and Kohn, 1964), calculations with Perdew-Burke-Ernzerhof (PBE) generalized gradient approximation (GGA) (Perdew et al., 1996a) predict pyrite to have a band gap of about 0.3 eV smaller than the experimental value of 0.95 eV as generally accepted (Ennaoui et al., 1993; Schena et al., 2013; Kolb and Kolpak, 2013; Li et al., 2015). Considering the well-known band gap problem of local density approximation (LDA) or GGA (Perdew et al., 1982), orbital-dependent functionals in spirit of generalized Kohn-Sham (GKS) DFT (Seidl et al., 1996; Perdew et al., 2017; Zhang et al., 2020) are also employed to tackle the problem, e.g. PBE plus the Hubbard-*U* correction (DFT + *U*) and hybrid functionals (Becke, 1993a; Becke, 1993b; Perdew et al., 1996b). Using an *ad hoc* *U* of 2 eV, the PBE + *U* method is able to reproduce the experimental band gap (Sun et al., 2011; Hu et al., 2012; Li et al., 2018) but meanwhile deteriorates the simulated optical spectra compared to PBE (Choi et al., 2012; Schena et al., 2013). Furthermore, despite the good performance in predicting band gaps for typical semiconducting materials (Heyd et al., 2005; Paier et al., 2006b,a; Marsman et al., 2008), hybrid functionals such as Heyd-Scuseria-Ernzerhof (HSE) method (Heyd et al., 2003, 2006) have been shown to give large band gaps for pyrite of over 2 eV (Muscat et al., 2002; Sun et al., 2011; Choi et al., 2012; Hu et al., 2012; Schena et al., 2013; Liu et al., 2019). There are also works using beyond-DFT methods, particularly, the *GW* method based on many-body perturbation theory (MBPT) (Hedin, 1965). However, the *GW* results for the pyrite phase are rather scattered, ranging from 0.3 to 1.1 eV (Choi et al., 2012; Lehner et al., 2012; Kolb and Kolpak, 2013; Schena et al., 2013). It is worth noting that Schena and coworkers conducted the state-of-the-art all-electron *G*_0_ *W*_0_ calculations with the linearized augmented plane-wave (LAPW) basis for both pyrite and marcasite, and report a pyrite band gap only about 0.3 eV (Schena et al., 2013). The *GW* gap value is smaller than that from PBE, which is rarely observed in *GW* practices and hence deserves closer investigation.

For *GW* implementations involving explicit summation of states, it is established recently by a number of works (Friedrich et al., 2006; Friedrich et al., 2011a; Friedrich et al., 2011b; Klimes et al., 2014; Jiang and Blaha, 2016; Nabok et al., 2016; Jiang, 2018; Zhang and Jiang, 2019; Ren et al., 2021) that an accurate description of high-lying empty states is essential to give accurate correlation self-energy operator and consequent QP band structure. In the pseudo-potential framework, one can improve the accuracy by using a norm-conserving potential with specifically tailored projectors at high energies (Klimes et al., 2014; van Setten et al., 2018). In all-electron calculations with the LAPW basis set, local orbitals with large energy parameters (usually 10^1∼2^ Ry higher than the Fermi level) are introduced as additional basis functions to remove the linearization error in unoccupied states up high in the conduction band regime (Friedrich et al., 2006, 2011a,b; Jiang and Blaha, 2016; Nabok et al., 2016). The LAPW basis extended by these high-energy local orbitals (HLOs), termed as LAPW + HLOs, has succeeded in helping produce accurate QP band structures in good agreement with experiment for a variety of semiconductors (Jiang and Blaha, 2016) including the conventionally challenging systems such as ZnO (Friedrich et al., 2011a; Friedrich et al., 2011b; Stankovski et al., 2011; Jiang and Blaha, 2016; Nabok et al., 2016), *d*/*f*-electron oxides (Jiang, 2018) and cuprous and silver halides (Zhang and Jiang, 2019). Particularly, the effects of including HLOs on the QP correction have been demonstrated quantitatively to be larger for states with stronger metal-*d* characters (Zhang and Jiang, 2019). For the FeS_2_ polymorphs with states of significant Fe-3*d* characters in both valence and low-energy conduction band regimes, *GW* with LAPW + HLOs is likely to give better description of the QP energies and dispersion relation than that with the standard LAPW basis.

A competitive alternative in the DFT framework to *GW* for band structure prediction is the doubly screened hybrid (DSH) functional method (Cui et al., 2018) in the category of hybrid functionals with system-dependent parameters (Zhang et al., 2020). Derived from a model dielectric function (Cappellini et al., 1993; Shimazaki and Yoshihiro, 2008), the exchange-correlation potential in DSH can be regarded as a further approximation to the Coulomb hole and screened exchange (COHSEX) approximation to the *GW* self-energy, and is able to capture both dielectric and metallic screening in the exchange interaction (Cui et al., 2018). It is shown that the DSH can evaluate band gaps of typical *sp* semiconductors with accuracy comparable to *GW* with the LAPW + HLOs basis while only at modest computational cost (Cui et al., 2018). Furthermore, the one-shot variant DSH0 can outperform fixed-parameter hybrid functionals for band gap predictions in a wide range of materials including narrow-gap semiconductors and transition metal mono-oxides (Cui et al., 2018; Liu et al., 2020). Hence we consider DSH as a hopeful approach to solve the FeS_2_ band gap puzzle within the GKS framework of DFT.

In the present work, we investigate the electronic band structures of the pyrite and marcasite polymorphs of FeS_2_ by applying the state-of-the-art all-electron *GW* method with the LAPW + HLOs basis. For comparison, we examine the results from *GW* with the standard LAPW basis as well. We also investigate the performances of several hybrid functionals, including PBE0 (Perdew et al., 1996b), HSE06 (Heyd et al., 2003, Heyd et al., 2006), screened-exchange-PBE hybrid functional (SX-PBE) (Bylander and Kleinman, 1990; Seidl et al., 1996) and DSH (Cui et al., 2018), in attempt to obtain insights into the failure of the conventional fixed-parameter functionals in predicting the band gap of FeS_2_.

## 2 Theory and Methods

### 2.1 The *GW* Method

The central task of the *GW* method is to solve the quasi-particle (QP) equation with the self-energy operator Σ in the frequency domain expressed as (Hedin, 1965)

$$\Sigma \left(r,r^{\prime };\omega \right)=\frac{i}{2\pi }\int_{-\infty }^{\infty }d\omega^{\prime }e^{i\omega^{\prime }\delta }G\left(r,r^{\prime };\omega +\omega^{\prime }\right)W\left(r^{\prime },r;\omega^{\prime }\right)$$ (1)

where *G* is the time-ordered Green’s function

$$G\left(r,r^{\prime };\omega \right)=\underset{nk}{\sum }\frac{\psi_{nk}\left(r\right)\psi_{nk}^{*}\left(r^{\prime }\right)}{\omega -\varepsilon_{nk}+i\eta sgn\left(\varepsilon_{nk}-\mu \right)}$$ (2)

With *ψ*_*n* **k**_ and *ε*_*n* **k**_ being the wave function and energy of the single-particle state $\left|nk\rangle \right\rangle$ respectively, *μ* the chemical potential, and *δ* and *η* positive infinitesimals. Atomic units are used throughout the paper. The screened Coulomb interaction *W* writes

$$W\left(r,r^{\prime };\omega \right)=\int dr^{\prime \prime }\varepsilon^{-1}\left(r,r^{\prime \prime };\omega \right)v\left(r^{\prime \prime },r^{\prime }\right)$$ (3)

where *v* (**r**, **r**′) = 1/|**r** − **r**′| is the bare Coulomb interaction and *ε*(**r**, **r**′; *ω*) is the microscopic dielectric function calculated at the level of random phase approximation (RPA). In principle, Eqs 1–3 have to be solved self-consistently along with the Dyson equation for the Green’s function (Hedin, 1965). However, due to the computational cost and generally unsatisfactory results of the fully self-consistent *GW* for solids [e.g. Grumet et al. (2018)], one usually turns to the non-self-consistent variant *G*_0_ *W*_0_. Considering the resemblance of KS and QP wave functions in weakly correlated systems (Hybertsen and Louie, 1986), the self-energy or QP energy $\varepsilon_{nk}^{QP}$ can be computed perturbatively upon the acquisition of Σ from the KS states as

$$\varepsilon_{nk}^{QP}=\varepsilon_{nk}^{KS}+Z_{nk}\left\langle \langle nk\right|\hat{\Sigma }\left(\varepsilon_{nk}^{KS}\right)-{\hat{V}}_{xc}|nk\rangle$$ (4)

where *V*_xc_ is the KS exchange-correlation potential and *Z*_*n* **k**_ a renormalization factor. One can further perform the so-called energy-only self-consistent *GW*_0_ calculations, where QP energies $\varepsilon_{nk}^{QP}$ in place of $\varepsilon_{nk}^{KS}$ in Eq. 2 are updated iteratively while *W* is kept the same as in *G*_0_ *W*_0_ (Shishkin and Kresse, 2007). The *GW* method has been implemented in various numerical frameworks (Jiang, 2011; Golze et al., 2019). For a detailed explanation of the basic theory and computational techniques used in the present *GW* implementation, the readers can refer to Jiang et al. (2013).

### 2.2 All-Electron Calculations With HLOs-Extended LAPW Basis

In the all-electron framework with LAPW, KS wave functions are expanded by the LAPW basis (Andersen, 1975; Singh and Nordström, 2006; Blaha et al., 2020)

$$\varphi_{k+G}^{\text{LAPW}}\left(r\right)=\left\{\begin{array}{cc} \frac{1}{\sqrt{V}}e^{i\left(k+G\right)\cdot r}\; & r\notin V_{\alpha }\\ \underset{lm}{\sum }\left[A_{\alpha lm}^{k+G}u_{\alpha l}\left(r^{\alpha };E_{\alpha l}\right)+B_{\alpha lm}^{k+G}{\dot{u}}_{\alpha l}\left(r^{\alpha };E_{\alpha l}\right)\right]Y_{l}^{m}\left({\hat{r}}^{\alpha }\right)\; & r\in V_{\alpha } \end{array}\right.$$ (5)

where *V*_*α*_ is the region enclosed by the muffin-tin (MT) sphere of atom *α* centered at **r**_*α*_ with radius $R_{MT}^{\alpha }$ , **r** ^*α*^ = **r** − **r**_*α*_, *u*_*αl*_ (*E*_*αl*_) is the solution of radial KS equation inside *V*_*α*_ at chosen energy *E*_*αl*_, ${\dot{u}}_{\alpha l}\left(E_{\alpha l}\right)\equiv {\left.\partial u_{\alpha l}\left(E\right)/\partial E\right|}_{E=E_{\alpha l}}$ , and $Y_{l}^{m}$ is the spherical harmonic function. The coefficients $A_{\alpha lm}^{k+G}$ and $B_{\alpha lm}^{k+G}$ are determined by enforcing that $\varphi_{k+G}^{\text{LAPW}}\left(r\right)$ be smooth at the boundary of *V*_*α*_. Local orbitals (LOs) which vanish outside the atomic spheres are proposed to supplement the LAPW basis to better describe the semi-core states (Singh, 1991). Inside the atomic sphere *V*_*α*_, LOs take the following form

$$\varphi_{\alpha lm}^{LO,i}\left(r\right)=\left[A_{\alpha lm}^{LO,i}u_{\alpha l}\left(r^{\alpha };E_{\alpha l}\right)+B_{\alpha lm}^{LO,i}{\dot{u}}_{\alpha l}\left(r^{\alpha };E_{\alpha l}\right)+C_{\alpha lm}^{LO,i}u_{\alpha l}\left(r^{\alpha };E_{\alpha l}^{LO,i}\right)\right]Y_{l}^{m}\left({\hat{r}}^{\alpha }\right)$$ (6)

where $E_{\alpha l}^{LO,i}$ is the energy parameter for the *i*th LO centered on atom *α* with angular and azimuthal quantum numbers *l* and *m*, respectively.

HLOs fall into the category of LOs with $E_{\alpha l}^{LO}$ typically 10 ∼ 100 Ry above the Fermi level. Such extra LOs have been found to facilitate accurate description of unoccupied states by remedying the linearization error therein when using the LAPW basis (Krasovskii et al., 1994; Krasovskii, 1997; Friedrich et al., 2006; Michalicek et al., 2013). In ground state calculations with LDA/GGA or hybrid functionals, the error causes no essential difficulties, since only occupied and low-lying unoccupied states are involved which are usually handled in sufficient accuracy with the usual or standard LAPW basis generated as default in popular DFT implementations with LAPW basis (Blaha et al., 2020). However, the error can be detrimental to the numerical accuracy of methods where the summation over unoccupied states is required, e.g. *GW* and DFT methods with density approximations belonging to the fifth rung of Jacobi ladder (Perdew and Schmidt, 2001) such as the adiabatic-connection dissipation-fluctuation (ACFD) calculation under RPA for ground-state energy (Ren et al., 2012; Cui et al., 2016; Zhang et al., 2018). In these methods, the completeness of summation and quality of unoccupied states play a crucial role. Previous *GW* studies (Jiang and Blaha, 2016; Jiang, 2018; Zhang and Jiang, 2019; Shen et al., 2020) have suggested that both can be taken into account by including localized orbitals energetically higher than the Fermi level in addition to the standard LAPW basis. HLOs have been shown to effectively improve the optical properties (Krasovskii et al., 1994; Krasovskii, 1997), NMR chemical shifts (Laskowski and Blaha, 2012; Laskowski and Blaha, 2014), *GW* QP energies (Friedrich et al., 2006; Friedrich et al., 2011a; Jiang and Blaha, 2016; Nabok et al., 2016; Jiang, 2018; Zhang and Jiang, 2019), optimized effective potential (Betzinger et al., 2011, 2012) and RPA correlation energy (Betzinger et al., 2015).

In the current implementation, HLOs are generated systematically by following the way described by Laskowski and Blaha (2012). The quality of LAPW + HLOs is controlled by two parameters besides those for the LAPW basis, namely, the additional number of nodes in the radial function of highest energy local orbital with respect to that of the LAPW function with the same angular quantum number and the maximum angular quantum number of used HLOs, denoted as *n*_LO_ and $l_{max}^{\left(LO\right)}$ , respectively. Generally speaking, the larger *n*_LO_ and $l_{max}^{\left(LO\right)}$ are, the higher the HLOs can reach in the energy space. We use *n*_LO_ = 0 to denote the usual or standard LAPW basis. Since the convergence rate of the QP energy with respect to the two parameters can be different for states featuring distinct atomic characters, careful convergence check is required to obtain numerically accurate *GW* results.

### 2.3 Hybrid Functionals

Hybrid functionals have been widely used in first-principles simulations of condensed matter for their good balance between performance and computational cost, and have been actively developed to further exploit the potential of its particular functional form. Readers interested in detailed description on the current status of hybrid functional development are directed to several recent reviews (Kümmel and Kronik, 2008; Baer et al., 2010; Maier et al., 2019; Zhang et al., 2020). Here we briefly introduce the general formalism of the range-separated hybrid functionals and the variants relevant to the current study.

The essential ingredient in hybrid functional methods is the exchange-correlation energy *E*_xc_ or potential *V*_xc_ composed of non-local orbital-dependent (screened) Hartree-Fock (HF) exchange terms. In the present work, we focus on hybrid functionals with *V*_xc_ in the range-separated form as (Zhang et al., 2020)

$$\begin{array}{cc} V_{xc}\left(x,x^{\prime }\right)= & \alpha_{sr}\left[V_{\text{x}}^{HF,sr}\left(x,x^{\prime };\mu \right)-V_{\text{x}}^{SL,sr}\left(x;\mu \right)\delta \left(x-x^{\prime }\right)\right]\\ & +\alpha_{lr}\left[V_{\text{x}}^{HF,lr}\left(x,x^{\prime };\mu \right)-V_{\text{x}}^{SL,lr}\left(x;\mu \right)\delta \left(x-x^{\prime }\right)\right]\\ & \;+V_{xc}^{SL}\left(x\right)\delta \left(x-x^{\prime }\right) \end{array}$$ (7)

where $V_{\text{x}}^{HF,sr}$ and $V_{\text{x}}^{HF,lr}$ are the short- and long-ranged Fock exchange potentials, respectively, which, using the reduced density-matrix defined as $\rho \left(x,x^{\prime }\right)\equiv \sum_{i\in \text{occ}}\psi_{i}\left(x\right)\psi_{i}*\left(x^{\prime }\right)$ , can be written as

$$\begin{array}{cc} V_{\text{x}}^{HF,sr}\left(x,x^{\prime };\mu \right) & =-\rho \left(x,x^{\prime }\right)v^{sr}\left(r,r^{\prime };\mu \right)\\ V_{\text{x}}^{HF,lr}\left(x,x^{\prime };\mu \right) & =-\rho \left(x,x^{\prime }\right)\left[v\left(r,r^{\prime }\right)-v^{sr}\left(r,r^{\prime };\mu \right)\right]. \end{array}$$ (8)

In Eq. 8 *v* ^sr^ (**r**, **r**′; *μ*) denotes the short-ranged Coulomb interaction of a certain form characterized by screening parameter *μ* (Zhang et al., 2020). **x** denotes collectively the spatial and spin coordinates of an electron, **x** ≡ (**r**, *σ*). $V_{\text{x}}^{SL,sr}$ and $V_{\text{x}}^{SL,lr}$ are the semi-local (SL) counterparts of the exchange potentials in LDA, GGA or meta-GGA. *μ* and the mixing ratios *α*_sr_ and *α*_lr_ are the adjustable parameters of the hybrid functional form.

Conventionally, the parameters are determined by either theoretical analysis or fitting against some dataset of particular properties, and then applied to other systems as fixed. Famous examples of the fixed-parameter hybrid functionals include PBE0 *α*_sr_ = *α*_lr_ = 1/4 (Perdew et al., 1996b) and the HSE series *α*_sr_ = 1/4, *α*_lr_ = 0, *μ* = 0.2–0.3 Å^-1^ (Heyd et al., 2003; Heyd et al., 2006). Recently, hybrid functionals with system-dependent parameters are developed by several groups (Shimazaki and Yoshihiro, 2008; Marques et al., 2011; Kronik et al., 2012; Koller et al., 2013; Skone et al., 2014; Chen et al., 2018; Cui et al., 2018). Among different methods, the doubly screened hybrid (DSH) functional has been demonstrated as a competitive candidate for accurate description of band structures of both wide- and narrow-gap semiconductors (Cui et al., 2018). The underlying idea of DSH is to approximate the screening effect in solids by employing the Bechstedt model dielectric function (Bechstedt et al., 1992)

$$\varepsilon \left(q\right)=1+\left[{\left(\varepsilon_{M}-1\right)}^{-1}+\alpha {\left(\frac{q}{q_{TF}}\right)}^{2}\right]$$ (9)

where *ɛ*_M_ is the macroscopic dielectric constant, *q*_TF_ the Thomas-Fermi wave vector and *α* an empirical parameter chosen for semiconductors (Cappellini et al., 1993). A screened Coulomb interaction can be derived from this model to take both dielectric and metallic screening into account, leading to parameters in Eq. 7 as

$$\alpha_{sr}=1,\;\alpha_{lr}=\frac{1}{\varepsilon_{M}},\;\mu =\frac{2q_{TF}}{3\sqrt{\alpha }}{\left(\frac{1}{\varepsilon_{M}-1}+1\right)}^{1/2}.$$ (10)

The corresponding short-ranged Coulomb interaction in Eq. 8 is

$$v^{sr}\left(r,r^{\prime };\mu \right)=\frac{erfc\left(\mu |r-r^{\prime }|\right)}{\left|r-r^{\prime }\right|}$$ (11)

where erfc is the complementary error function. In practice, an initial *ɛ*_M_ is required, which can be obtained from the PBE calculation or experimental measurements, to construct the DSH potential and solve the GKS equation. The resulting single-particle states act as the inputs to compute a new *ɛ*_M_, which is in turn used to update the DSH potential. The self-consistent loop stops when *ɛ*_M_ is converged. Alternatively, one can break after solving the GKS equation with the initial *ɛ*_M_, leading to the one-shot scheme denoted as DSH0.

### 2.4 Computational Details

**FIGURE 1:**
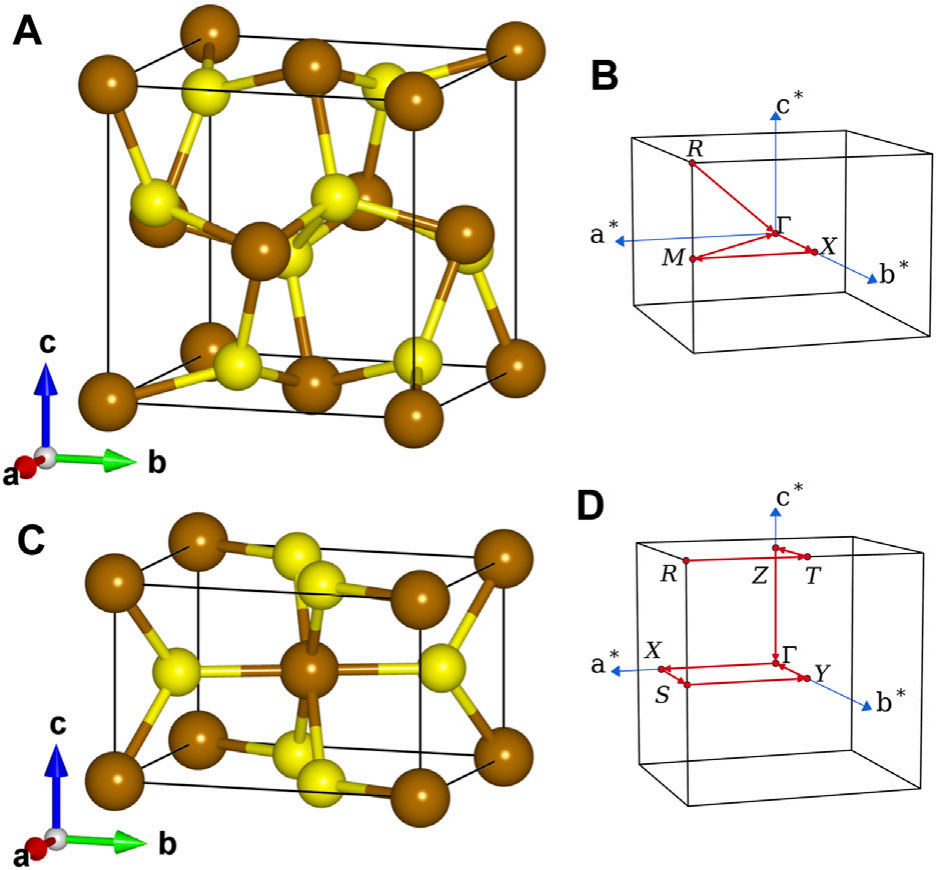
Lattice structures **(A,C)** and first Brillouin zones **(B,D)** of pyrite **(upper)** and marcasite **(lower)** phases of FeS_2_. Brown and yellow spheres represent Fe and S atoms, respectively.

The unit cells of pyrite and marcasite FeS_2_ used in our calculations are shown in the left panel of Figure 1. The crystal structure of pyrite FeS_2_ (Figure 1A) can be viewed as a faced-centered cubic cell of Fe atoms with S_2_ dumbbells occupying the octahedral interstitials and pointing to different <111> crystallographic axes. The anion coordination octahedra (FeS6) are connected only through sharing vertices. In the orthorhombic marcasite phase (Figure 1C), (FeS6) are connected by sharing edges with the two neighbors along *c*-axis and linked together through sharing vertices on the *aOb* plane. In terms of lattice parameters, we use *a* = 5.418 Å, *u* = 0.3850 for pyrite (space group $Pa\bar{3}$ ) and *a* = 4.443 Å, *b* = 5.425 Å, *c* = 3.387 Å, *u* = 0.2005, *v* = 0.3783 for marcasite (space group *Pnnm*). These values follow the results from X-ray diffraction experiments at ambient conditions (Brostigen and Kjekshus, 1969; Brostigen et al., 1973; Chattopadhyay and Von Schnering, 1985; Zuñiga-Puelles et al., 2019). The corresponding S-S bond lengths in the two polymorphs are 2.16 and 2.21 Å, respectively.

The present all-electron *GW* calculations are performed by the *GW* facilities in the GAP2 program (Jiang et al., 2013; Jiang and Blaha, 2016) interfaced to WIEN2k (Blaha et al., 2001, 2020). Results in both *G*_0_ *W*_0_ and *GW*_0_ schemes are presented, where KS orbital energies and wave functions calculated with the PBE (Perdew et al., 1996a) GGA are used as the input to construct one-body Green’s function and screened Coulomb interaction. The KS states are obtained by using charge density pre-converged under self-consistent field (SCF) calculation with PBE and the standard LAPW basis. The energy criterion for convergence of SCF iterations is set to 10^–8^ Rydberg (Ry). 64 (4 × 4 × 4) and 120 (5 × 4 × 6) **k** points are sampled in the first Brillouin zones of pyrite and marcasite FeS_2_, respectively. All available unoccupied states are considered in the summation of states for screened Coulomb interaction *W* and self-energy Σ. Mixed product basis is used to describe the wave function products in the two-point functions, e.g. *W* and Σ (Aryasetiawan and Gunnarsson, 1994; Kotani and van Schilfgaarde, 2002). We choose *Q* = 0.75 and $l_{\text{max}}^{\text{MB}}=3$ for the interstitial plane wave and MT product basis, respectively [Jiang et al. (2013) for the meanings of these parameters]. LAPW and LOs with *E*_*αl*_ < 20 Ry are used to build the MT product basis. Frequency dependence of *W* is treated explicitly on a 16-point double Gauss-Legendre grids along the positive imaginary axis. Σ on the same grid is calculated and analytically continued to the real axis (Rojas et al., 1995). A rather coarse **k**/**q**-point mesh, 2 × 2 × 2 for pyrite and 4 × 2 × 4 for marcasite, is sufficient to converge the direct band gap at the Γ point ${E_{g}}^{\Gamma }$ within 0.01 eV. The QP band structure diagrams along particular **k**-point paths (see the right panel of Figure 1) are calculated by interpolating the QP energies obtained with the above mesh using the Fourier interpolation technique (Pickett et al., 1988).

In terms of the LAPW basis, the usual or standard LAPW basis set is created automatically in the recent version of WIEN2k (Blaha et al., 2001), which is actually a mixture of the APW + lo basis for the valence states (Madsen et al., 2001), the ordinary LAPW basis for higher *l* channels up to *l*_max_ = 10 and additional local orbitals (LOs) for semi-core Fe-3*s* and Fe-3*p* states (Blaha et al., 2001). The convergence with respect to the two HLOs parameters *n*_LO_ and $l_{max}^{\left(LO\right)}$ is investigated, the latter being represented by $\Delta l_{LO}=l_{max}^{\left(LO\right)}-l_{max}^{\left(v\right)}$ where $l_{max}^{\left(v\right)}$ is the largest *l* of valence orbitals for each element. In the present study, $l_{max}^{\left(v\right)}=2$ and $l_{max}^{\left(v\right)}=1$ for Fe and S, respectively. Since the convergence with respect to HLOs parameters are decoupled from the choice of **k**-point mesh, we choose marcasite with a coarse 2 × 1 × 2 mesh for HLOs convergence test. *RKmax* ≡ *R*_MT,min_ *K*_max_ = 7.0 is chosen for the plane-wave cut-off in the interstitial region, where *R*_MT,min_ is the minimal muffin-tin radius *R*_MT_ used in the lattice. In the present FeS_2_ case, *R*_MT_ is set to 2.1 Bohr for Fe and 1.9 Bohr for S. Using *RKmax* = 9.0 will reduce the band gap from *GW* (LAPW + HLOs) by less than 0.03 eV, indicating that adequate accuracy can be delivered with the current *RKmax* = 7.0 setup. Due to limited computational resources, *RKmax* = 6.0 is used for HLOs convergence test. Following Laskowski and Blaha (2012), the linear independence of HLO basis functions is assured by choosing the energy parameters such that the overlap between the HLO radial functions is smaller than a threshold, which is 0.6 in the present work.

For hybrid functional calculations, we consider PBE0 (Perdew et al., 1996b), HSE06 (Heyd et al., 2003, 2006) and screened exchange SX-PBE (Bylander and Kleinman, 1990) methods as well as DSH. All hybrid functional calculations are performed with the projector augmented waves (PAW) method (Blöchl, 1994) implemented in the Vienna *ab-initio* Simulation Package (VASP) (Kresse and Furthmüller, 1996). The static dielectric function is calculated from the average of diagonal elements of macroscopic dielectric tensor computed by using density functional perturbation theory (DFPT) with local field effect included (Baroni et al., 2001). Apart from 3*d* and 4*s*, the 3*s* and 3*p* electrons of Fe are also treated explicitly in the valence region. The Thomas-Fermi wave vectors are 2.57 and 2.56 Å^-1^ for pyrite and marcasite FeS_2_, respectively. The cut-off energy of plane-wave basis for wave function expansion is chosen as 400 eV, which is sufficient to converge ${E_{g}}^{\Gamma }$ of both FeS_2_ polymorphs within 2 meV. In terms of **k**-point mesh, 64 (4 × 4 × 4) and 120 (5 × 4 × 6) **k** points are sampled in the first Brillouin zones of pyrite and marcasite for the self-consistent calculations, respectively. Using a finer 6 × 6 × 6 sampling for pyrite will change the band gap by less than 0.01 eV, and hence we consider the results well converged with respect to the **k**-point mesh. The energy convergence criterion is chosen to be 10^–6^ eV for the SCF iterations.

## 3 Results and Discussion

### 3.1The *GW* Results

In this part, we present the electronic band structures of pyrite and marcasite FeS_2_ computed by the all-electron *GW* method. In particular, we analyse the effect of high-energy local-orbitals (HLOs) by comparing the results from *GW* with the standard LAPW and LAPW + HLOs basis.

#### 3.1.1 Convergence of QP Energies with Respect to HLOs Parameters

To achieve a balance between the computational cost and numerical accuracy of the LAPW + HLOs based *GW* method, we have to decide an optimized HLOs setup for the FeS_2_ polymorphs of interest. That is to say, certain convergence with respect to the two HLOs parameters, namely *n*_LO_ and Δ*l*_LO_, must be achieved for the QP band structures of both polymorphs, while the number of basis functions should be kept as few as possible. To simplify the notation, we denote the setup of HLOs by (*n*_LO_, Δ*l*_LO_) so that (1, 1) indicates a set of HLOs with *n*_LO_ = 1 and Δ*l*_LO_ = 1, for example. Since we are most interested in the band gaps (direct and indirect) of the systems, we choose the indirect band gap from the *X* point to the Γ point, $E_{g}^{\text{X–}\Gamma }$ , as the descriptor for the band structure, and investigate its dependence on the two HLOs parameters for marcasite.

Before discussing the results, we briefly illustrate the appropriateness of this choice. First of all, $E_{g}^{\text{X–}\Gamma }$ is a representative band gap energy for pyrite and marcasite FeS_2_. This is because in both phases, the topmost valence state at the *X* point, *X*_v_, is close to the valence band maximum (VBM) and the bottommost conduction state at the Γ point, Γ_c_, is the conduction band minimum (CBM) (that is the case for marcasite given the coarse 2 × 1 × 2 **k** mesh in the convergence study). Second, either *X*_v_ or Γ_c_ has similar atomic contributions in the two polymorphs, and the effects of HLOs on such states are also similar, as shown in the results for other polymorphs like zinc-blende and wurtzite ZnO (Jiang and Blaha, 2016). Therefore the parameters optimized for marcasite are considered transferable and can be applied to the pyrite polymorph. Last but not least, as we will discuss later, the effects of HLOs on *X*_v_ and Γ_c_ differ significantly, avoiding considerable error cancellation in change of the QP correction to the band gap upon including HLOs.

**FIGURE 2:**
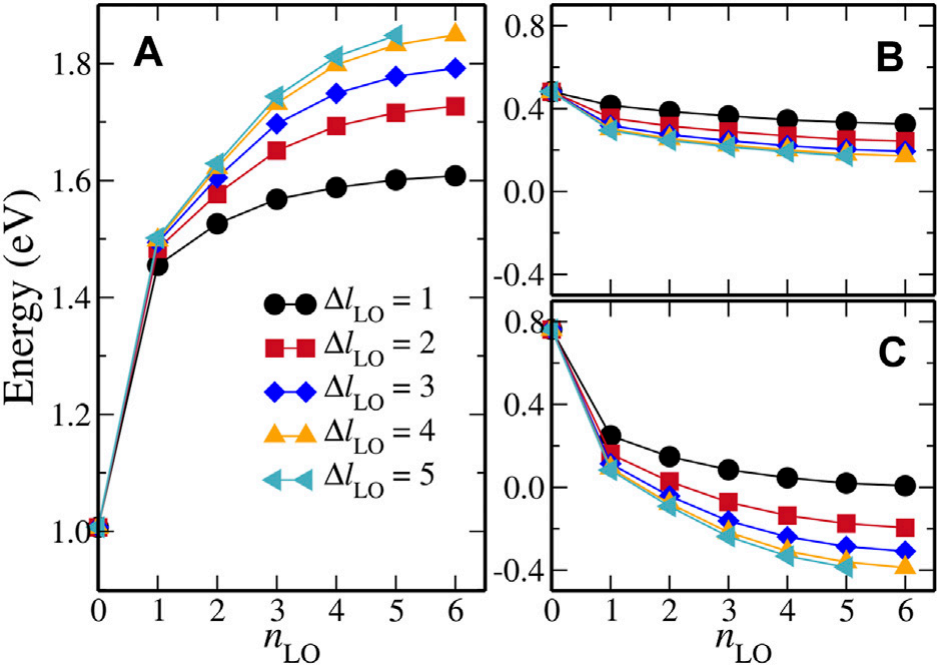
Dependence on HLOs parameters of *G*_0_ *W*_0_@PBE calculated **(A)** the indirect band gap between *X* and Γ, $E_{g}^{\text{X–}\Gamma }$ , and self-energy corrections to **(B)** bottommost conduction state at the Γ point, $\Delta \epsilon_{\text{c}}^{\Gamma }$ , and **(C)** topmost valence state at the *X* point, $\Delta \epsilon_{\text{v}}^{X}$ , for marcasite FeS_2_.

Figure 2 summarizes the results of convergence study for the *G*_0_ *W*_0_@PBE method. $E_{g}^{\text{X–}\Gamma }$ (Figure 2A) is about 1.0 eV with the standard LAPW basis (*n*_LO_ = 0) and is significantly increased by extending LAPW with HLOs. One can see that the convergence rate of $E_{g}^{\text{X–}\Gamma }$ with respect to *n*_LO_ differs with different Δ*l*_LO_, and is faster for lower Δ*l*_LO_. The reverse is also true, i.e. the convergence with respect to Δ*l*_LO_ is faster when *n*_LO_ is smaller. It clearly indicates that the convergence with respect to *n*_LO_ and Δ*l*_LO_ is coupled. Increasing HLOs parameters from (4, 4) to (5, 5) changes $E_{g}^{\text{X–}\Gamma }$ by less than 0.05 eV, indicating that HLOs (4,4) is able to deliver an adequate accuracy. Therefore, unless stated otherwise, HLOs (4, 4), amounting to 196 and 145 HLOs for Fe and S atoms, respectively, is considered optimized and will be used in the subsequent *GW* calculations denoted by LAPW + HLOs. The energy parameters for HLOs (4, 4) can be found in Table 1.

**TABLE 1:** Energy parameters (unit: Rydberg) of high-energy local orbitals (HLOs) used in pyrite FeS_2_ corresponding to HLOs parameters *n*_LO_ = 4, Δ*l*_LO_ = 4 for Fe and S. Those for marcasite are essentially the same with difference by 0.02 Ry at most in each element and *l* channel.

*l* =	0	1	2	3	4	5	6
S	5.30	15.72	14.46	24.02	33.08	42.40	—
—	35.76	34.72	30.82	43.64	55.88	68.30	—
—	62.42	59.66	53.00	69.00	84.30	99.70	—
—	95.04	90.32	80.96	99.94	118.24	136.60	—
—	133.50	—	—	—	—	—	—
Fe	18.72	19.68	8.15	18.22	26.44	34.42	42.66
—	38.14	38.46	20.29	33.16	44.28	55.00	65.86
—	62.80	62.28	37.37	52.92	66.72	80.06	93.46
—	92.48	90.90	59.25	77.40	93.78	109.62	125.50

It is worth noting that the effect of including HLOs on the QP correction to $E_{g}^{\text{X–}\Gamma }$ is different from those on the valence and conduction states. To illustrate this, we show the dependence on *n*_LO_ and Δ*l*_LO_ of the self-energy corrections to Γ_c_ ( $\Delta \varepsilon_{\text{c}}^{\Gamma }$ ) and *X*_v_ ( $\Delta \varepsilon_{\text{v}}^{X}$ ) states in Figures 2B,C, respectively. Both $\Delta \varepsilon_{\text{c}}^{\Gamma }$ and $\Delta \varepsilon_{\text{v}}^{X}$ decrease with increasing *n*_LO_ or Δ*l*_LO_, but the former converges much faster than the latter, which agrees with the general trend observed previously (Jiang and Blaha, 2016; Zhang and Jiang, 2019). With the standard LAPW basis, *G*_0_ *W*_0_@PBE gives $\Delta \varepsilon_{\text{c}}^{\Gamma }=0.48$ eV and $\Delta \varepsilon_{\text{v}}^{X}=0.76$ eV, indicating a negative QP correction to the band gap, which is rarely observed in LDA/GGA-based *GW* calculations of semiconductors (Jiang and Blaha, 2016). When HLOs (5, 5) are included, $\Delta \varepsilon_{\text{c}}^{\Gamma }$ decreases by 0.3 eV, much smaller compared to the decreasing of 1.2 eV in $\Delta \varepsilon_{\text{v}}^{X}$ . Such biased effects of including HLOs on valence and conduction band states can be attributed to the difference in atomic characteristics between the states, and will be further discussed in the following sections.

#### 3.1.2 Quasi-Particle Band Gaps

After having obtained the optimized HLOs, we perform the PBE-based *GW* calculations for pyrite and marcasite FeS_2_ with the LAPW + HLOs basis set, and compare with the PBE method and *GW* with the standard LAPW basis.

The band gaps of pyrite and marcasite FeS_2_ calculated by PBE and *GW* methods are presented in Table 2. The fundamental band gaps are obtained by computing the band energies along the **k**-point paths indicated in the right panel of Figure 1. In the PBE reference, pyrite and marcasite are predicted to have indirect fundamental band gaps of 0.70 and 0.83 eV, respectively. Our PBE results are consistent with those from previous all-electron LAPW study (Schena et al., 2013) and close to the recently reported optical band gaps obtained from diffuse reflectance spectroscopy (Sánchez et al., 2016). However, our PBE band gap for pyrite is slightly larger than several reported PBE results (Sun et al., 2011; Kolb and Kolpak, 2013; Lazić et al., 2013; Li et al., 2015; Zhang et al., 2018). This can be attributed to the use of different lattice structures in those studies (Eyert et al., 1998; Lazić et al., 2013; Schena et al., 2013) from the current work. Particularly, geometry optimization by PBE (Eyert et al., 1998; Schena et al., 2013) generally gives a longer S-S dimer, which leads to smaller splitting between bonding and anti-bonding S-3*pσ* orbitals and a consequent shrink in the band gap.

**TABLE 2:** Fundamental band gap (indicated by “fund.”) and other direct and indirect band gaps (unit: eV) for pyrite and marcasite FeS_2_ calculated by PBE and *GW* methods. Results from previous *GW* studies and experimental measurements are presented for comparison. To simplify the notation, we use “L” and “L + H” to denote the standard LAPW and LAPW + HLOs basis sets, respectively. PBE is used as the starting point for *G*_0_ *W*_0_ and *GW*_0_ calculations unless stated otherwise.

Methods	Pyrite					Marcasite				
	Fund.	Γ → Γ	*X* → Γ	*X* → *X*	*M* → Γ	Fund.	Γ → Γ	Γ → *T*	*X* → Γ	*X* → *T*
PBE	0.70	0.82	0.72	1.68	0.85	0.83	1.74	1.37	1.32	0.95
*G*_0_ *W*_0_ (L)	0.06	0.11	0.08	1.96	0.32	0.57	0.88	1.62	0.80	1.53
*GW*_0_ (L)	metal	—	—	—	—	0.29	0.60	1.62	0.59	1.61
*G*_0_ *W*_0_ (L + H)	1.04	1.16	1.06	2.14	1.18	1.15	1.80	1.55	1.54	1.28
*GW*_0_ (L + H)	1.14	1.28	1.16	2.21	1.28	1.16	1.87	1.56	1.59	1.28
Previous *GW*	—	—	—	—	—	—	—	—	—	—
$G_{0}W_{0}$ ^a^	—	0.28	0.31	1.67	—	1.06	1.40	—	1.19	1.40
$G_{0}W_{0}$ ^b^	—	0.61	0.63	1.72	—	—	1.88	—	1.57	1.25
$GW_{0}$ ^c^	0.97	—	—	—	—	—	—	—	—	—
sc*GW* ^d^	1.01	—	—	—	—	—	—	—	—	—
QS*GW* ^e^	0.81	—	—	—	—	—	—	—	—	—
Expt	0.95^f^, 0.82^g^	—	—	—	—	0.83^g^	—	—	—	—

a From Schena et al. (2013), using LAPW extended by HLOs up to 800 eV and with Fe 3*s*, 3*p* LOs included.

b From Schena et al. (2013), using LAPW extended by HLOs up to 800 eV but without Fe 3*s*, 3*p* LOs.

c From Ouarab and Boumaour (2017).

d From Kolb and Kolpak (2013), using PAW method and experimental lattice constants.

e From Lehner et al. (2012), using LMTO method.

f From Ennaoui et al. (1993).

g From Sánchez et al. (2016), optical gap at room temperature using diffuse reflectance spectroscopy.

For *GW* calculations with the standard LAPW basis, the QP fundamental band gaps by *G*_0_ *W*_0_@PBE are smaller than the PBE counterparts in both FeS_2_ polymorphs. Pyrite FeS_2_ is predicted to have a band gap of only 0.06 eV, which is 0.64 eV smaller than that by PBE. The negative QP correction for pyrite band gap has been reported by Schena et al. (2013). The QP fundamental band gap for marcasite predicted by *G*_0_ *W*_0_@PBE (LAPW) is also smaller than PBE, while the change (0.26 eV) is less dramatic than that for pyrite. Such negative QP corrections to LDA/GGA band gaps are uncommon in *GW* studies for closed-shell systems (Klimes et al., 2014; Jiang and Blaha, 2016; van Setten et al., 2017; Zhang and Jiang, 2019) as well as open-shell *d*/*f*-electron semiconductors (Jiang, 2018). Switching on self-consistency of the Green’s function by *GW*_0_@PBE further reduces the fundamental band gaps of FeS_2_. In particular, pyrite is predicted to be metallic by *GW*_0_@PBE, which disagrees qualitatively with its semiconducting nature in experiment (Ennaoui et al., 1993). For other direct and indirect band gaps, those for Γ → Γ and *X* → Γ in pyrite and marcasite and *M* → Γ in marcasite are decreased from PBE to *G*_0_ *W*_0_@PBE (LAPW). The decrease is largest for the Γ → Γ gap in the two phases, 0.71 and 0.86 eV for pyrite and marcasite, respectively. On the other hand, the gaps for *X* → *X* in pyrite, Γ → *T* and *X* → *T* in marcasite are increased by 0.28, 0.25, and 0.58 eV, respectively. However, it should be noted that the distinction in signs of corrections to the QP gaps in different channels should not be considered as intrinsic for FeS_2_. Instead, it is an artifact as a result of the incomplete basis, which we will discuss in details below.

Now we turn to the LAPW + HLOs-based *GW* calculations. With the *G*_0_ *W*_0_@PBE method, including HLOs increases the QP fundamental gap by 0.98 eV for pyrite and 0.58 eV for marcasite. The resulting *G*_0_ *W*_0_@PBE band gaps are 1.04 and 1.15 eV for pyrite and marcasite, respectively. In contrast, all band gaps investigated are increased by *G*_0_ *W*_0_ with LAPW + HLOs compared to their PBE counterparts. We note that HLOs have distinct effects among band gaps for different channels. Once the HLOs are included, band gaps for channels with the conduction state at the Γ point are increased by about 1 eV. On the other hand, the QP correction to the *X* → *X* band gap in pyrite increases by only 0.18 eV. Moreover, the gaps for Γ → *T* and *X* → *T* in marcasite even decrease. With the LAPW + HLOs basis, using *GW*_0_ to switch on partial self-consistency further increases the band gaps, but the change is moderate and no more than 0.1 eV.

As explained at the beginning, the fundamental band gap of FeS_2_ has been controversial in the recent decades, partly due to the widely varying experimental values (Ennaoui et al., 1993). In the present study, the *GW*_0_@PBE method with the LAPW + HLOs predicts that pyrite and marcasite have indirect fundamental band gaps of 1.14 and 1.16 eV, respectively. The *GW*_0_ gap of pyrite is slightly larger than the generally accepted experimental value of 0.95 eV (Ennaoui et al., 1993). Furthermore, the fact that the two polymorphs have almost identical band gaps is consistent with the optical measurements by Sánchez et al. (2016), although our predicted band gaps are about 0.3 eV larger. However, it should be noted that one must take exciton binding energy *E*_B_ into account for a meaningful comparison between the QP fundamental band gap and experimentally measured optical gap. The difference between the fundamental and optical gaps can be significant when the exciton is localized, i.e. of Frenkel type (Fox, 2010). On the other hand, while it is more straightforward to compare the QP gap with spectral data from direct and inverse PES (Folkerts et al., 1987; Mamiya et al., 1997), the resolutions of available measurements for pyrite FeS_2_ are too low to extract a meaningful gap value for comparison. Moreover, to the best knowledge of the authors, no data of combined PES/IPS measurements are available for marcasite. Therefore, further experimental studies are required to determine and verify the band gaps of the FeS_2_ polymorphs.

To close this part, we highlight that the present work resolve two issues reported in previous *GW* studies in terms of QP band structures of FeS_2_. First, Schena et al. (2013) performed a *G*_0_ *W*_0_@PBE study on pyrite and marcasite FeS_2_ with similar HLOs-extended LAPW basis. The fundamental band gap of pyrite was estimated as about 0.3 eV, by which the authors claimed to explain the low *V*_OC_ encountered in the pyrite solar cell. However, according to our convergence study, such a small band gap is likely to result from inadequate convergence with respect to HLOs. More specifically, the largest angular momentum of HLOs $l_{max}^{\left(LO\right)}$ used in Schena et al. (2013) is 3, i.e. *f* orbital, while $l_{max}^{\left(LO\right)}=2+4=6$ (*i* orbital) is used in the optimized HLOs of the present work. As a result, the highest energy covered by HLOs in Schena et al. (2013) (800 eV) is much smaller than that used in the present work (about 1800 eV). Second, fully self-consistent *GW* (sc*GW*) and quasi-particle self-consistent *GW* (QPsc*GW* or QS*GW*) calculations have also been carried out to study the band structure of pyrite, and give apparently satisfactory results (Lehner et al., 2012; Kolb and Kolpak, 2013). However, variants of self-consistent *GW* without taking the vertex function into account tend to overestimate the band gaps of typical semiconductors, as indicated by several works (Shishkin and Kresse, 2007; Deguchi et al., 2016; Cao et al., 2017; Grumet et al., 2018). Thus the error cancellation between the general tendency of overestimating band gaps of semiconductors and the numerical inaccuracy in the LAPW basis or the use of conventional pseudo-potentials could contribute to the apparent agreement between the generally accepted band gap and the self-consistent *GW* results. Of course, without looking into computational details of previous self-consistent *GW* calculations, this is just our speculation. Further investigations are needed to fully clarify this issue. We also note that similar LAPW + HLOs calculation has been conducted for the pyrite phase by Ouarab and Boumaour (2017) and gives a band gap (0.97 eV) close to ours.

#### 3.1.3 Quasi-Particle Band Structure

To further illustrate the significance of HLOs in applying the *GW* methods to FeS_2_, we present the QP band structures of pyrite and marcasite FeS_2_ calculated from the *G*_0_ *W*_0_@PBE method with the LAPW + HLOs basis set, and compare the results to those with the standard LAPW basis.

**FIGURE 3:**
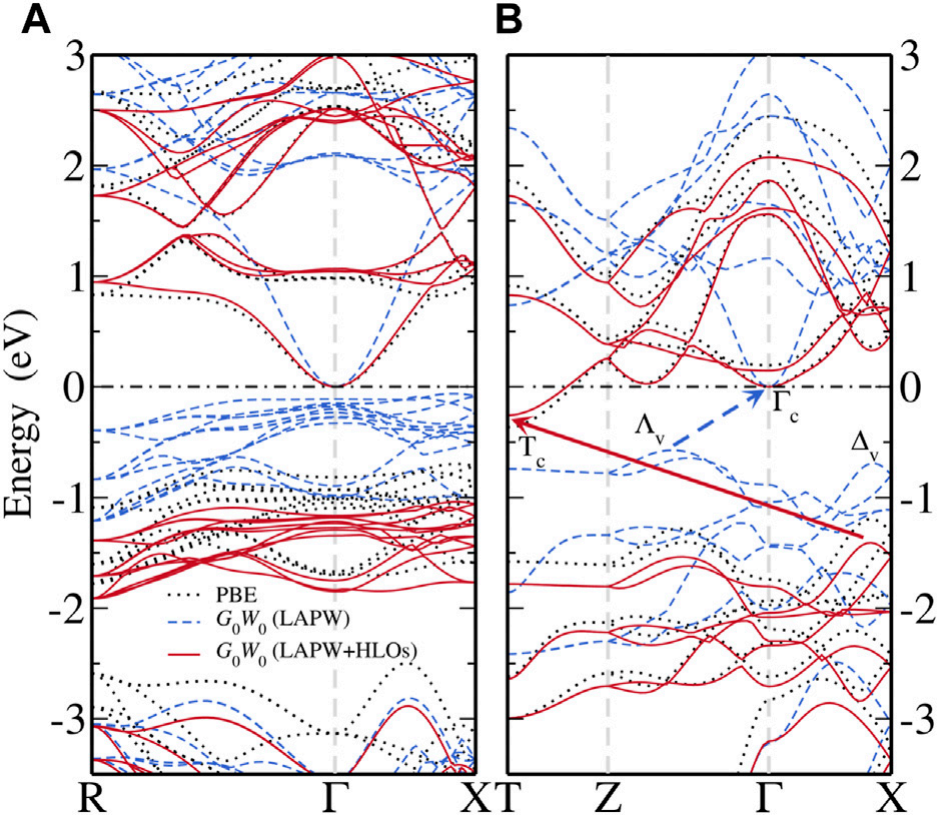
Comparison of band structures computed from PBE (black dotted), *G*_0_ *W*_0_@PBE with the standard LAPW basis (blue dashed) and *G*_0_ *W*_0_@PBE with the LAPW basis extended by optimized HLOs (LAPW + HLOs, red solid) for **(A)** pyrite and **(B)** marcasite FeS_2_. The conduction band minimum is aligned as the energy zero and marked by the black dash-dotted line.

**FIGURE 4:**
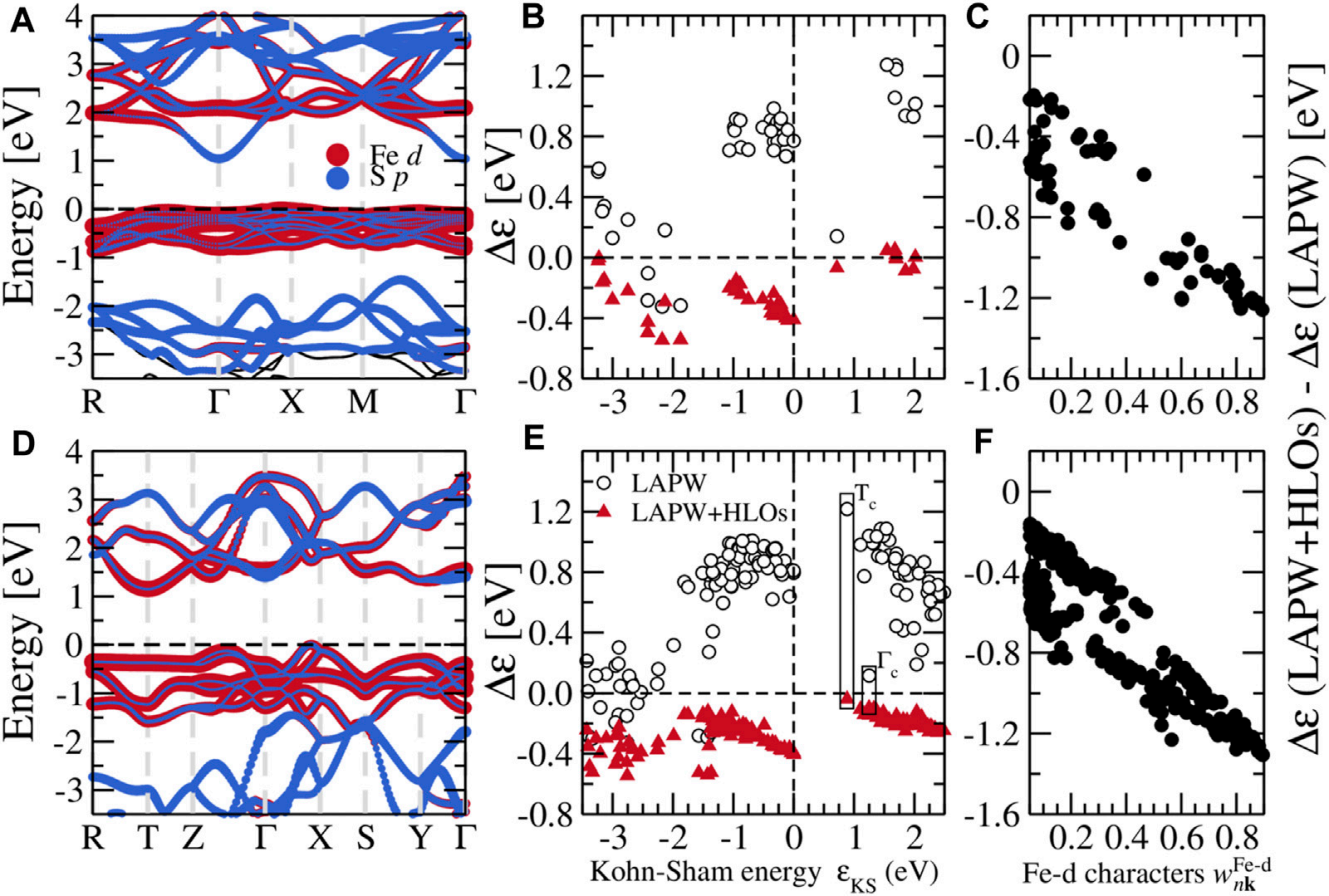
**(A)** Projected band structure from *GW* with the LAPW + HLOs basis, **(B)** self-energy corrections Δ*ɛ* to Kohn-Sham states, and **(C)** the difference between Δ*ɛ* with LAPW + HLOs and LAPW basis against the weight of Fe-*d* characters $w_{nk}^{Fe-d}$ defined by Eq. 12 for pyrite FeS_2_. *GW* are performed at the *G*_0_ *W*_0_ level. The quasi-particle **(A)** and Kohn-Sham **(B)** energies are aligned to the corresponding valence band maximum. In **(A)**, projections of states on Fe-*d* and S-*p* orbitals are proportional to the diameters of red and blue circles, respectively. **(D–F)** are the counterparts for the marcasite phase.

Figure 3 shows the electronic band structures of the two FeS_2_ phases from different methods. Note that the bands are aligned to the CBM at the Γ point for a better view of QP correction to the valence states. With PBE, pyrite (Figure 3A) is found to be an indirect band gap material with the CBM located at the Γ point and the VBM near the *X* point along Γ–*X*. The top valence bands within 1 eV below the VBM are dominated by the localized Fe-3*d* states, also manifested by their flat dispersion. The dispersive bands about 2 eV below the VBM are mainly composed of S-3*p* states and well separated from the Fe-3*d* (*t*_2*g*_) valence bands. In the conduction band region, the lowermost conduction bands are also largely composed of Fe-3*d* (*e*_*g*_), except for the states close to the Γ point with predominant S-3*p* characters. Particularly, the CBM Γ_c_ state is exclusively formed by the *σ* anti-bonding overlapping of S-3*p* orbitals in the S-S dimer (see projected bands in Figure 4A). Valence and conduction bands with strong Fe-3*d* characters are separated by about 2 eV. For marcasite, an indirect band gap is also observed, with the CBM located at the *T* point (*T*_c_) and the VBM along Γ–*X* (Δ_v_). Both states at the VBM and CBM of marcasite are of dominant Fe-3*d* characters (Figure 4D), in contrast to pyrite where CBM is of pure S-3*p* characters. The wider Fe-3*d* valence bands overlap with the S-3*p* bands near about 1.5 eV below the VBM, which indicates stronger covalent bonding between Fe and S in marcasite than in pyrite.

Then we compare the QP band structures obtained from *G*_0_ *W*_0_@PBE with the LAPW and LAPW + HLOs basis (Figure 3). With the standard LAPW basis, *G*_0_ *W*_0_@PBE predicts pyrite almost as a semimetal with a nearly vanishing band gap (Figure 3A). Dispersion of the conduction band around the Γ point and the separation between the Fe-3*d* and S-3*p* valence bands are enhanced compared to the PBE reference. For marcasite (Figure 3B), although a noticeable gap (0.57 eV) is predicted by *G*_0_ *W*_0_@PBE, the band edges are different from those in PBE: the CBM is located at the Γ point (Γ_c_) and the VBM in the middle of the *Z*–Γ path (Λ_v_). The change in the nature of band edges from semi-local functional to *GW* method is also observed by Schena et al. (2013). Once HLOs are included in the basis set, QP band gaps of both phases are dramatically enlarged. The fundamental gaps of pyrite and marcasite are 1.04 and 1.15 eV, respectively, which are 0.2 ∼ 0.3 eV larger than the optical gaps from absorption spectra (Sánchez et al., 2016). Band edges of marcasite by *GW* are also recovered to those by PBE. The comparison indicates that both negative QP corrections to band gaps and change of band edges in *GW* (LAPW) are indeed artifacts due to the inadequate numerical accuracy of the basis set.

To better understand how the HLOs basis functions influence the QP band structures of FeS_2_, we scrutinize the QP correction to Kohn-Sham state Δ*ɛ*, defined by the difference between the QP energy *ɛ*_QP_ and the KS energy *ɛ*_KS_, i.e. Δ*ɛ* ≡*ɛ*_QP_ − *ɛ*_KS_. For pyrite, with the standard LAPW basis, Δ*ɛ* to the CBM is smaller than those to the valence Fe-3*d t*_2*g*_ and conduction Fe-3*d e*_*g*_ states as shown in Figure 4B. Particularly, Δ*ɛ* for the VBM is about 0.7 eV greater than that for the CBM. This leads to a up-shift of Fe-3*d* states with respect to the CBM on a whole. Extending LAPW with HLOs reduces Δ*ɛ* for all states, but the reduction in Δ*ɛ* to the VBM is more than that to the CBM by about 1.0 eV, resulting in the sign change of the QP correction to the band gap. Similar conclusion can be drawn from Δ*ɛ* in the marcasite phase (Figure 4E). With the standard LAPW method, Δ*ɛ* to *T*_c_ exceeds that to Γ_c_ by more than 1.1 eV. Consequently, Γ_c_ drops down below *T*_c_ and becomes the CBM, as we have seen in Figure 3B. Upon including HLOs, Δ*ɛ* to *T*_c_ is reduced more significantly than Δ*ɛ* to Γ_c_ such that *T*_c_ recovers the conduction band edge as in PBE.

Such biased effects of HLOs are clearly associated with the atomic characteristics of Kohn-Sham states, as we have demonstrated in the *GW* calculations of cuprous and silver halides (Zhang and Jiang, 2019). In Figures 4C,F, we plot the difference between Δ*ɛ* computed by *G*_0_ *W*_0_ with LAPW + HLOs and LAPW against the weight of Fe-*d* characters of the Kohn-Sham orbitals $\left|\psi_{nk}\rangle \right\rangle$ , $w_{nk}^{Fe-d}$ , defined by

$$w_{nk}^{Fe-d}=\underset{i}{\sum }\overset{2}{\underset{m=-2}{\sum }}{\left|\left\langle \phi_{l=2,m}^{{\text{Fe}}_{i}}\left.\right|\psi_{nk}\right\rangle \right|}^{2}$$ (12)

where $\phi_{l=2,m}^{{\text{Fe}}_{i}}$ represents the pre-defined atomic function centered on the *i*th Fe atom featuring spherical harmonic function $Y_{2}^{m}$ . The negative difference implies that including HLOs generally brings down Δ*ɛ*. Moreover, the difference is more dramatic for states with larger $w_{nk}^{Fe-d}$ , indicating that numerical error is more significant for states with stronger Fe-*d* characters in *GW* calculations with the incomplete LAPW basis.

#### 3.1.4 *GW* Density of States

**FIGURE 5:**
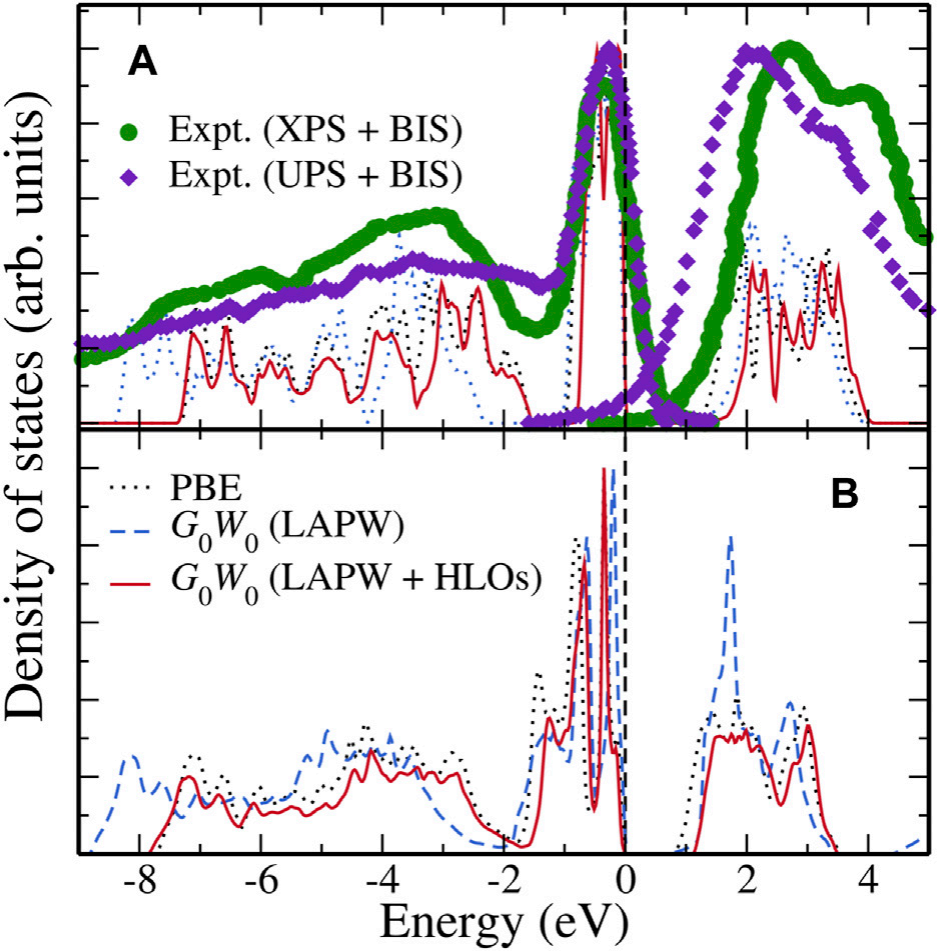
Density of states (DOS) computed from PBE (black dotted), *G*_0_ *W*_0_@PBE with the standard LAPW basis (blue dashed) and *G*_0_ *W*_0_@PBE with the LAPW + HLOs basis (red solid) for **(A)** pyrite and **(B)** marcasite FeS_2_. Energy distribution curves (EDCs) of pyrite extracted from photo-electron spectroscopy (PES) are presented for comparison. Each dataset is normalized with respect to its highest peak. Theoretical data are aligned to its valence band maximum as energy zero. The XPS + BIS and UPS + BIS data for pyrite are obtained from Folkerts et al. (1987) and Mamiya et al. (1997), respectively. To take into account the different definitions of the Fermi level in theory and experiment, a rigid shift of 0.60 and 0.40 eV are employed for the EDCs from XPS + BIS and UPS + BIS, respectively, to match the highest valence peaks below the Fermi level.

To end this section, we present the *GW* calculated density of states (DOS) of FeS_2_ polymorphs in Figure 5. The results for pyrite FeS_2_ are shown in Figure 5A. Due to different definitions of the Fermi level in theoretical results and experimental spectral data, we have shifted the experimental data to match up the highest valence peak near the Fermi level. With this alignment, the overall DOS from *G*_0_ *W*_0_ (LAPW + HLOs) agrees well with the energy distribution curves (EDCs) from the PES experiments. The width of the valence Fe-3*d* band and separation between the Fe-3*d* and S-3*p* valence bands are consistent with the UPS experiment by Mamiya et al. (1997) and the XPS experiment by Folkerts et al. (1987). The location of the first peak in the conduction band region is also in good agreement with the BIS data (Folkerts et al., 1987). Interestingly, although *G*_0_ *W*_0_ (LAPW) underestimates the band gap severely, the location of the first peak in the conduction region is almost identical to that by *G*_0_ *W*_0_ (LAPW + HLOs), probably due to the error cancellation between QP corrections to the valence and conduction Fe-3*d* bands. However, such fortuitous cancellation does not hold in the valence region as inferred by the too deep S-3*p* band in the *G*_0_ *W*_0_ (LAPW) results.

Figure 5B shows the calculated DOS for marcasite. Regardless of the theoretical method used, the valence Fe-3*d* band of marcasite has larger width than that of pyrite, indicating a stronger Fe-S interaction in the marcasite phase. In the conduction region, a sharp peak is observed with the *G*_0_ *W*_0_ (LAPW) method, while only a plateau is found with *G*_0_ *W*_0_ (LAPW + HLOs). However, the sharp peak is actually an artifact of wrongly pushed up Fe-3*d* conduction bands due to the inaccuracy of the standard LAPW basis as explained above.

### 3.2 Results From Hybrid Functionals

As mentioned in the introduction, previous theoretical studies found that various hybrid functionals, which are typically able to describe the band gaps of semiconductors quite accurately, performed badly for FeS_2_. In this section, we look into this issue and present results by several hybrid schemes including the DSH functional with system-dependent parameters.

#### 3.2.1 Band Gaps by Hybrid Functionals

Band gaps computed by different hybrid functionals are collected in Table 3. The widely used PBE0 and HSE06 functionals have been reported to predict fundamental gaps of pyrite and marcasite FeS_2_ larger than 2 eV in the literature (Sun et al., 2011; Choi et al., 2012; Hu et al., 2012; Schena et al., 2013; Liu et al., 2019), which is confirmed by our results. DSH, the hybrid functional with system-tuned parameters, does not improve the prediction over PBE0 and HSE06. This is surprising, given that DSH has been previously shown to outperform several other hybrids in evaluating band structures for wide- and narrow-gap systems (Cui et al., 2018; Liu et al., 2020), including PBE0, HSE06 and the dielectric-dependent hybrid (DDH) functionals (Marques et al., 2011; Skone et al., 2014). SX-PBE screened exchange functional gives band gaps of FeS_2_ significantly smaller than the hybrids mentioned above, but the gaps are still larger than those from *GW* with the LAPW + HLOs basis (Table 2) by about 0.5 eV.

**TABLE 3:** Fundamental band gap (indicated by “fund.”) and other direct and indirect band gaps (unit: eV) for pyrite and marcasite FeS_2_ calculated by different hybrid functionals. Results from other theoretical studies and experimental measurements are presented as comparison.

Methods	Pyrite					Marcasite				
	Fund.	Γ → Γ	*X* → Γ	*X* → *X*	*M* → Γ	Fund.	Γ → Γ	Γ → *T*	*X* → Γ	*X* → *T*
PBE0	2.94	2.94	3.04	4.34	3.03	2.95	3.63	4.01	3.22	3.60
HSE06	2.22	2.22	2.32	3.58	2.31	2.26	2.91	3.26	2.53	2.88
MHSE	1.16	1.29	1.18	2.32	1.27	1.47	2.09	1.96	1.72	1.59
DSH0^a^	2.43	2.87	2.70	4.06	2.55	2.16	3.49	4.25	2.59	3.35
DSH0^b^	2.72	3.19	3.02	4.39	2.85	—	—	—	—	—
DSH^c^	2.96	3.46	3.28	4.67	3.10	2.57	3.90	4.69	3.00	3.79
SX-PBE	1.69	1.74	1.82	3.20	1.73	1.64	3.08	3.00	2.30	2.21
HSE06^d^	2.76	—	—	—	—	2.72	—	—	—	—
HSE06^e^	2.69	—	—	—	—	—	—	—	—	—
HSE06^f^	2.70	—	—	—	—	—	—	—	—	—
HSE06^g^	2.2	—	—	—	—	—	—	—	—	—
HSE06^h^	2.40	—	—	—	—	2.16	—	—	—	—
MHSE^h^	1.14	—	—	—	—	1.26	—	—	—	—
PBE0^h^	2.76	—	—	—	—	2.94	—	—	—	—
Expt.	0.95^i^, 0.82^j^	—	—	—	—	0.83^j^	—	—	—	—

a Using ${\varepsilon_{M}}^{\text{PBE}}$ calculated by finite field method.

b Using *ɛ*_M_ = 10.9 obtained from Husk and Seehra (1978).

c Converged *ɛ*_M_: pyrite 7.8 and marcasite 9.2.

d From Sun et al. (2011), using PAW with experimental lattice constants.

e From Hu et al. (2012), using PAW with optimized lattice parameters (*a* = 5.422 Å, *u* = 0.385).

f From Choi et al. (2012), using PAW with experimental lattice constants.

g From Schena et al. (2013), using LAPW with optimized lattice parameters (*a* = 5.403 Å, *u* = 0.383).

h From Liu et al. (2019), using PAW.

i From Ennaoui et al. (1993).

j From Sánchez et al. (2016), optical gap at room temperature using diffuse reflectance spectroscopy.

Considering that the one-shot DSH, i.e. DSH0, may outperform the self-consistent scheme in some transition metal compounds (Cui et al., 2018; Liu et al., 2020), we also employ DSH0 to calculate the two FeS_2_ polymorphs. The macroscopic dielectric constant calculated with PBE ${\varepsilon_{M}}^{\text{PBE}}$ is 20.6 for pyrite, which agrees well with ${\varepsilon_{M}}^{\text{PBE}}=21$ from a previous study (Choi et al., 2012). DSH0 with ${\varepsilon_{M}}^{\text{PBE}}$ predicts smaller band gaps than DSH, but the values are still above 2 eV. Meanwhile, DSH0 with experimentally obtained *ɛ*_M_ = 10.9 (Husk and Seehra, 1978) gives the pyrite band gap of 2.72 eV. In contrast, a modified HSE functional (MHSE) with HSE06 screening parameter and 10% hybrid ratio, which is roughly equal to the inverse of the experimental dielectric constant, as suggested by Liu et al. (2019), gives band gaps close to the *GW*_0_ (LAPW + HLOs) result. The MHSE results agree with those by Liu et al. (2019) and seem to verify the suggestion by Schena et al. (2013) of using 1/*ɛ*_M_ as the hybrid ratio in the HSE-type screened hybrid functional.

#### 3.2.2 Band Structures by Hybrid Functionals

As summarized above, the investigated hybrid functionals except for MHSE fail to give reasonable predictions for the band gaps of pyrite and marcasite FeS_2_. In this section, we take a close look at the band structures computed from these methods to understand the failure.

**FIGURE 6:**
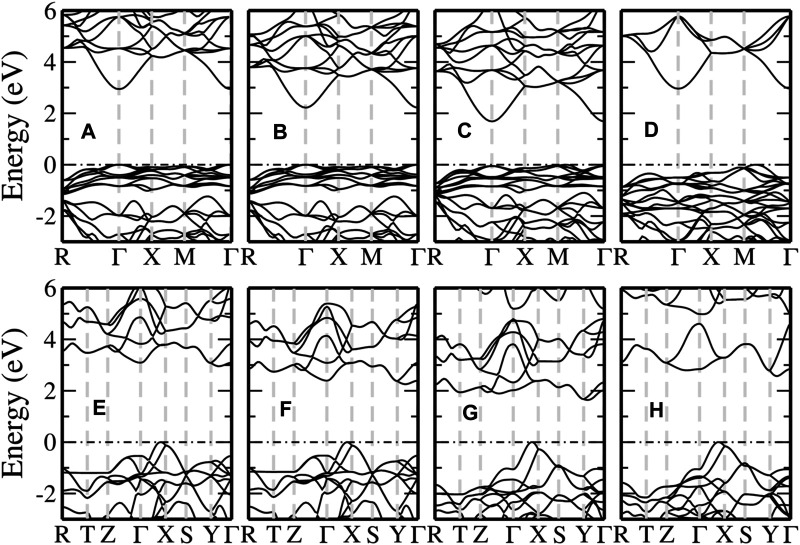
Band structures of pyrite **(upper panel)** and marcasite **(lower panel)** FeS_2_ computed from different hybrid functional methods. From left to right, the methods used are PBE0 **(A,E)**, HSE06 **(B,F)**, SX-PBE **(C,G)** and DSH **(D,H)**, respectively. The valence band maximum is aligned as the energy zero and indicated by the black dash-dotted line.

The band structures for pyrite calculated from selected hybrid functionals are shown in the upper panel of Figure 6. With PBE0 and HSE06 (Figures 6A,B), the fundamental band gap is a direct one with both VBM and CBM located at the Γ point. An indirect fundamental gap is obtained by SX-PBE and DSH (Figures 6C,D), but the VBM is different from that in PBE or the *GW* method (Figure 3A). In addition, compared to the *GW* (LAPW + HLOs) results, the separation between valence Fe-3*d* and S-3*p* bands is reduced and the splitting between the valence and conduction Fe-3*d* bands is significantly increased by the hybrid functionals. We note that both features can be understood tentatively as a result of increased ligand field strength from the perspective of ligand field theory. This indicates an overestimated interaction between the ligand S-3*pσ* and Fe-3*d* orbitals in the selected hybrid functionals than that in PBE. The overestimation is most significant in the DSH method (Figure 6D), where the state of predominant S-3*pπ* characters along the *M*–Γ path becomes the VBM and conduction Fe-3*d* bands are raised beyond 6 eV above the Fermi level.

We can observe similar features in marcasite band structures from hybrid functionals, as shown in the lower panel of Figure 6. In the valence band region, the S-3*p* bands are pushed up relatively to Fe-3*d* bands compared with PBE and *GW*. The increase is so significant that the VBM along Γ–*X*, which is mainly of Fe-3*d* in PBE and *GW*, is now of predominant S-3*p* characters. This also leads to a considerable overlap between the two sets of bands in the energy window 1 ∼ 3 eV below the Fermi level. The conduction bands are also shifted to higher energies. However, the shifts are larger for the conduction Fe-3*d* bands than for S-3*p*. For the DSH method as an extreme case, the Fe-3*d* bands are raised up too high and even separated from the S-3*p* bands.

The radical failure of DSH invites a close inspection of feasibility of DSH for FeS_2_. As a preliminary exploration to the possible cause, we make a direct comparison between the inverse static dielectric function used in the DSH with ${\varepsilon_{M}}^{\text{PBE}}$ and that from the RPA calculation with the LAPW + HLOs basis in pyrite FeS_2_ as a function of the length of wave vector in the long-range limit, i.e. *q* → 0. The inverse dielectric function corresponding to DSH reads (Liu et al., 2020)

$$\varepsilon_{\text{DSH}}^{-1}\left(G\right)=1-\left(1-\frac{1}{\varepsilon_{M}}\right)\left(e^{-{\left|G\right|}^{2}/4\mu^{2}}\right).$$ (13)

**FIGURE 7:**
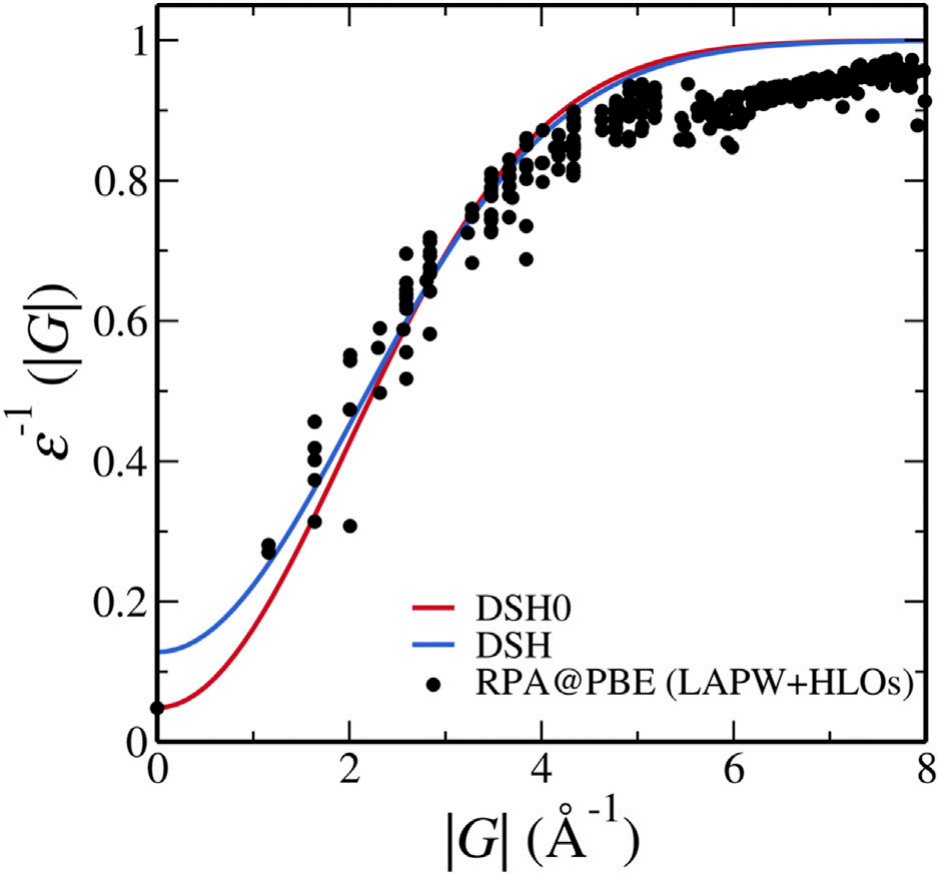
Inverse dielectric functions of pyrite used in the DSH model and calculated from RPA@PBE with the LAPW + HLOs basis set. Dielectric constant computed by PBE is used in the one-shot DSH0 method.

We note that Eq. 13 differs from the inverse of Eq. 9 because in the derivation of DSH, the exponential function is replaced by erfc [Cui et al. (2018) for more details]. As shown in Figure 7, while DSH overestimates *ɛ* ^−1^ and underestimates the screening in the short-wavelength region, i.e. near |**G**| = 0, DSH0 model dielectric function with ${\varepsilon_{M}}^{\text{PBE}}$ closely resembles that from RPA calculation, which is similar to the observation by Liu et al. (2020) in transition metal oxides. Hence we consider that the screening effect is reasonably captured in DSH0. Further investigation is needed to understand the cause for the failure of DSH for FeS_2_.

## 4 Conclusion

In the present study, we have investigated the electronic band structures of two FeS_2_ polymorphs, namely pyrite and marcasite, by using methods in different frameworks. With the all-electron many-body *GW* method implemented in the LAPW framework, we find that by using *GW*_0_@PBE with the LAPW + HLOs basis, pyrite and marcasite are predicted to have indirect fundamental band gaps of 1.14 and 1.16 eV, respectively. The closeness of band gaps for the two polymorphs agrees with the experimental observation (Sánchez et al., 2016). The pyrite band gap from *GW*_0_@PBE with LAPW + HLOs is very close to the generally accepted experimental value (Ennaoui et al., 1993) and the corresponding density of states also agrees well with energy distribution curves obtained from the photoelectron spectroscopy measurements (Folkerts et al., 1987; Mamiya et al., 1997). In contrast, with the standard LAPW basis, PBE-based *G*_0_ *W*_0_ and *GW*_0_ both lead to negative QP correction to the PBE fundamental gap, which is rarely observed in LDA/GGA-based *G*_0_ *W*_0_ and *GW*_0_ treatments of semiconductors. The splitting between Fe-3*d* and S-3*p* valence bands of pyrite is also significantly overestimated compared to experiment. These artifacts exist not only in calculations with the standard LAPW basis, but also in those with LAPW basis extended by an inadequately converged HLOs (Schena et al., 2013). Therefore in order to eliminate such artifacts, it is instrumental to carefully converge the fundamental band gap with respect to the two controlling parameters, namely *n*_LO_ and Δ*l*_LO_. We have further studied electronic band structures of FeS_2_ polymorphs with different hybrid functionals, including PBE0, HSE06, the screened exchange SX-PBE and the recently developed DSH functional with system-tuned hybridization parameters. We find that all those methods overestimate the band gaps of the two polymorphs by 0.5 ∼ 1.9 eV compared to the results obtained from *G*_0_ *W*_0_ (LAPW + HLOs). The overestimation by PBE0 and HSE06 as reported in the literature is reproduced in this work. Furthermore, either self-consistent or one-shot DSH method fails to improve over the conventional fixed-parameter hybrid functionals. By comparing the model dielectric function used in DSH with that from RPA calculation with LAPW + HLOs in pyrite, we point out that the failure of DSH may not be caused by the insufficiency of the dielectric model used and therefore requires further investigation. Our investigations clearly show that accurate prediction of electronic band structures of FeS_2_ polymorphs poses a stringent test on the state-of-the-art first-principles approaches, and the *GW* method based on semi-local density approximation performs well for this difficult system if it is practiced with well-converged numerical accuracy.

Finally, we note that further work in the following aspects can be done to shed more light onto the band gap problem of FeS_2_ in terms of *GW* and hybrid functional calculations. For one thing, it is possible to build the screened Coulomb interaction *W* using the KS states from the LAPW calculations and calculate the self-energy Σ with *G* from LAPW + HLOs. One can compare it with *GW* using LAPW to see whether it is the inaccurate band summation in *W* or *G* to blame. For another, replacing the PBE with the hybrid functional as starting point will be worthwhile to evaluate the dependence of *G*_0_ *W*_0_/*GW*_0_ results on initial input for FeS_2_. Particularly, considering the severe overestimation of FeS_2_ band gaps by the hybrid functionals, it is of great interest to see whether *G*_0_ *W*_0_/*GW*_0_ can produce a negative QP correction to the gap from hybrid functional calculations such that the experimental gap is approached from above.

## Data Availability

The original contributions presented in the study are included in the article/Supplementary Material, further inquiries can be directed to the corresponding author.

## References

[B1] Andersen O. K. (1975). Linear Methods in Band Theory. Phys. Rev. B 12, 3060–3083. 10.1103/physrevb.12.3060

[B2] Aryasetiawan F. Gunnarsson O. (1994). Product-basis Method for Calculating Dielectric Matrices. Phys. Rev. B 49, 16214–16222. 10.1103/physrevb.49.16214 10010768

[B3] Baer R. Livshits E. Salzner U. (2010). Tuned Range-Separated Hybrids in Density Functional Theory. Annu. Rev. Phys. Chem. 61, 85–109. 10.1146/annurev.physchem.012809.103321 20055678

[B4] Barawi M. Ferrer I. J. Flores E. Yoda S. Ares J. R. Sánchez C. (2016). Hydrogen Photoassisted Generation by Visible Light and an Earth Abundant Photocatalyst: Pyrite (FeS2). J. Phys. Chem. C 120, 9547–9552. 10.1021/acs.jpcc.5b11482

[B5] Baroni S. de Gironcoli S. Dal Corso A. Giannozzi P. (2001). Phonons and Related crystal Properties from Density-Functional Perturbation Theory. Rev. Mod. Phys. 73, 515–562. 10.1103/revmodphys.73.515

[B6] Bechstedt F. Del Sole R. Cappellini G. Reining L. (1992). An Efficient Method for Calculating Quasiparticle Energies in Semiconductors. Solid State. Commun. 84, 765–770. 10.1016/0038-1098(92)90476-p

[B7] Becke A. D. (1993a). A New Mixing of Hartree-Fock and Local Density‐functional Theories. J. Chem. Phys. 98, 1372–1377. 10.1063/1.464304

[B8] Becke A. D. (1993b). Density‐functional Thermochemistry. III. The Role of Exact Exchange. J. Chem. Phys. 98, 5648–5652. 10.1063/1.464913

[B9] Betzinger M. Friedrich C. Blügel S. Görling A. (2011). Local Exact Exchange Potentials within the All-Electron Flapw Method with Pseudopotential Results and a Comparison. Phys. Rev. B 83, 045105. 10.1103/physrevb.83.045105

[B10] Betzinger M. Friedrich C. Görling A. Blügel S. (2015). Precise All-Electron Dynamical Response Functions: Application to Cohsex and the Rpa Correlation Energy. Phys. Rev. B 92, 245101. 10.1103/physrevb.92.245101

[B11] Betzinger M. Friedrich C. Görling A. Blügel S. (2012). Precise Response Functions in All-Electron Methods: Application to the Optimized-Effective-Potential Approach. Phys. Rev. B 85, 245124. 10.1103/physrevb.85.245124

[B12] Birkholz M. Fiechter S. Hartmann A. Tributsch H. (1991). Sulfur Deficiency in Iron Pyrite (FeS2−x) and its Consequences for Band-Structure Models. Phys. Rev. B 43, 11926–11936. 10.1103/physrevb.43.11926 9996968

[B13] Blaha P. Schwarz K. Madsen G. K. H. Kvasnicka D. Luitz J. (2001). WIEN2K, an Augmented Plane Wave + Local Orbitals Program For Calculating Crystal Properties (Karlheinz Schwarz, Techn. Universität Wien, Austria).

[B14] Blaha P. Schwarz K. Tran F. Laskowski R. Madsen G. K. H. Marks L. D. (2020). WIEN2k: An APW+lo Program for Calculating the Properties of Solids. J. Chem. Phys. 152, 074101. 10.1063/1.5143061 32087668

[B15] Blöchl P. E. (1994). Projector Augmented-Wave Method. Phys. Rev. B 50, 17953–17979. 10.1103/physrevb.50.17953 9976227

[B16] Bronold M. Pettenkofer C. Jaegermann W. (1994). Surface Photovoltage Measurements on Pyrite (100) Cleavage Planes: Evidence for Electronic Bulk Defects. J. Appl. Phys. 76, 5800–5808. 10.1063/1.358393

[B17] Brostigen G. Kjekshus A. Astrup E. E. Nordal V. Lindberg A. A. Craig J. C. (1969). Redetermined crystal Structure of FeS2 (Pyrite). Acta Chem. Scand. 23, 2186–2188. 10.3891/acta.chem.scand.23-2186

[B18] Brostigen G. Kjekshus A. Rømming C. Gronowitz S. Koskikallio J. Swahn C.-G. (1973). Compounds with the Marcasite Type crystal Structure. VIII. Redetermination of the Prototype. Acta Chem. Scand. 27, 2791–2796. 10.3891/acta.chem.scand.27-2791

[B19] Bylander D. M. Kleinman L. (1990). Good Semiconductor Band Gaps with a Modified Local-Density Approximation. Phys. Rev. B 41, 7868–7871. 10.1103/physrevb.41.7868 9993089

[B20] Cabán-Acevedo M. Kaiser N. S. English C. R. Liang D. Thompson B. J. Chen H.-E. (2014). Ionization of High-Density Deep Donor Defect States Explains the Low Photovoltage of Iron Pyrite Single Crystals. J. Am. Chem. Soc. 136, 17163–17179. 10.1021/ja509142w 25399991

[B21] Cao H. Yu Z. Lu P. Wang L.-W. (2017). Fully Converged Plane-Wave-Based Self-Consistent *GW* Calculations of Periodic Solids. Phys. Rev. B 95, 035139. 10.1103/physrevb.95.035139

[B22] Cappellini G. Del Sole R. Reining L. Bechstedt F. (1993). Model Dielectric Function for Semiconductors. Phys. Rev. B 47, 9892–9895. 10.1103/physrevb.47.9892 10005065

[B23] Chattopadhyay T. Von Schnering H. G. (1985). High Pressure X-ray Diffraction Study on P-FeS2, M-FeS2 and MnS2 to 340 Kbar: A Possible High Spin-Low Spin Transition in MnS2. J. Phys. Chem. Sol. 46, 113–116. 10.1016/0022-3697(85)90204-5

[B24] Chatzitheodorou G. Fiechter S. Könenkamp R. Kunst M. Jaegermann W. Tributsch H. (1986). Thin Photoactive FeS2 (Pyrite) Films. Mater. Res. Bull. 21, 1481–1487. 10.1016/0025-5408(86)90088-7

[B25] Chen W. Miceli G. Rignanese G.-M. Pasquarello A. (2018). Nonempirical Dielectric-dependent Hybrid Functional with Range Separation for Semiconductors and Insulators. Phys. Rev. Mater. 2, 073803. 10.1103/physrevmaterials.2.073803

[B26] Choi S. G. Hu J. Abdallah L. S. Limpinsel M. Zhang Y. N. Zollner S. (2012). Pseudodielectric Function and Critical-point Energies of Iron Pyrite. Phys. Rev. B 86, 115207. 10.1103/physrevb.86.115207

[B27] Cui Z.-H. Wang Y.-C. Zhang M.-Y. Xu X. Jiang H. (2018). Doubly Screened Hybrid Functional: An Accurate First-Principles Approach for Both Narrow- and Wide-gap Semiconductors. J. Phys. Chem. Lett. 9, 2338–2345. 10.1021/acs.jpclett.8b00919 29669414

[B28] Cui Z.-H. Wu F. Jiang H. (2016). First-principles Study of Relative Stability of Rutile and Anatase Tio_2_ Using the Random Phase Approximation. Phys. Chem. Chem. Phys. 18, 29914–29922. 10.1039/c6cp04973g 27761539

[B29] Deguchi D. Sato K. Kino H. Kotani T. (2016). Accurate Energy Bands Calculated by the Hybrid Quasiparticle Self-consistentGWmethod Implemented in the Ecalj Package. Jpn. J. Appl. Phys. 55, 051201. 10.7567/jjap.55.051201

[B30] Ennaoui A. Fiechter S. Jaegermann W. Tributsch H. (1986). Photoelectrochemistry of Highly Quantum Efficient Single‐Crystalline N ‐ FeS2 (Pyrite). J. Electrochem. Soc. 133, 97–106. 10.1149/1.2108553

[B31] Ennaoui A. Fiechter S. Pettenkofer C. Alonso-Vante N. Büker K. Bronold M. (1993). Iron Disulfide for Solar Energy Conversion. Solar Energ. Mater. Solar Cell 29, 289–370. 10.1016/0927-0248(93)90095-k

[B32] Eyert V. Höck K.-H. Fiechter S. Tributsch H. (1998). Electronic Structure ofFeS2: The Crucial Role of Electron-Lattice Interaction. Phys. Rev. B 57, 6350–6359. 10.1103/physrevb.57.6350

[B33] Ferrer I. J. Nevskaia D. M. de las Heras C. Sánchez C. (1990). About the Band gap Nature of FeS2 as Determined from Optical and Photoelectrochemical Measurements. Solid State. Commun. 74, 913–916. 10.1016/0038-1098(90)90455-k

[B34] Folkerts W. Sawatzky G. A. Haas C. Groot R. A. d. Hillebrecht F. U. (1987). Electronic Structure of Some 3d Transition-Metal Pyrites. J. Phys. C: Solid State. Phys. 20, 4135–4144. 10.1088/0022-3719/20/26/015

[B35] Fox M. (2010). Optical Properties of Solids. second edn. Oxford: OUP.

[B36] Friedrich C. Müller M. C. Blügel S. (2011a). Band Convergence and Linearization Error Correction of All-Electron *GW* Calculations: The Extreme Case of Zinc Oxide. Phys. Rev. B 83, 081101(R). 10.1103/physrevb.83.081101

[B37] Friedrich C. Müller M. C. Blügel S. (2011b). Erratum: Band Convergence and Linearization Error Correction of All-Electron GW Calculations: The Extreme Case of Zinc Oxide. Phys. Rev. B 83, 081101(r). 2011*Phys. Rev. B* 84, 039906. 10.1103/physrevb.83.081101

[B38] Friedrich C. Schindlmayr A. Blügel S. Kotani T. (2006). Elimination of the Linearization Error in GW Calculations Based on the Linearized Augmented-Plane-Wave Method. Phys. Rev. B 74, 045104. 10.1103/physrevb.74.045104

[B39] Golze D. Dvorak M. Rinke P. (2019). The GW Compendium: A Practical Guide to Theoretical Photoemission Spectroscopy. Front. Chem. 7, 377. 10.3389/fchem.2019.00377 31355177 PMC6633269

[B40] Gong M. Kirkeminde A. Kumar N. Zhao H. Ren S. (2013a). Ionic-passivated FeS2 Photocapacitors for Energy Conversion and Storage. Chem. Commun. 49, 9260–9262. 10.1039/c3cc45088k 23995298

[B41] Gong M. Kirkeminde A. Xie Y. Lu R. Liu J. Wu J. Z. (2013b). Iron Pyrite (FeS2) Broad Spectral and Magnetically Responsive Photodetectors. Adv. Opt. Mater. 1, 78–83. 10.1002/adom.201200003

[B42] Goodenough J. B. (1972). Energy Bands in TX2 Compounds with Pyrite, Marcasite, and Arsenopyrite Structures. J. Solid State. Chem. 5, 144–152. 10.1016/0022-4596(72)90022-9

[B43] Grumet M. Liu P. Kaltak M. Klimeš J. Kresse G. (2018). Beyond the Quasiparticle Approximation: Fully Self-Consistent GW Calculations. Phys. Rev. B 98, 155143. 10.1103/physrevb.98.155143

[B44] Hedin L. (1965). New Method for Calculating the One-Particle Green's Function with Application to the Electron-Gas Problem. Phys. Rev. 139, A796–A823. 10.1103/physrev.139.a796

[B45] Herbert F. W. Krishnamoorthy A. Van Vliet K. J. Yildiz B. (2013). Quantification of Electronic Band gap and Surface States on FeS2(100). Surf. Sci. 618, 53–61. 10.1016/j.susc.2013.08.014

[B46] Heyd J. Peralta J. E. Scuseria G. E. Martin R. L. (2005). Energy Band Gaps and Lattice Parameters Evaluated with the Heyd-Scuseria-Ernzerhof Screened Hybrid Functional. J. Chem. Phys. 123, 174101. 10.1063/1.2085170 16375511

[B47] Heyd J. Scuseria G. E. Ernzerhof M. (2003). Hybrid Functionals Based on a Screened Coulomb Potential. J. Chem. Phys. 118, 8207–8215. 10.1063/1.1564060

[B130] Heyd J. Scuseria G. E. Ernzerhof M. (2006). Erratum: “Hybrid Functionals Based on a ScreenedCoulomb Potential” [j. chem. phys.118, 8207 (2003)]. J. Chem. Phys. 124, 219906.

[B48] Hohenberg P. Kohn W. (1964). Inhomogeneous Electron Gas. Phys. Rev. 136, B864–B871. 10.1103/physrev.136.b864

[B49] Hu J. Zhang Y. Law M. Wu R. (2012). First-principles Studies of the Electronic Properties of Native and Substitutional Anionic Defects in Bulk Iron Pyrite. Phys. Rev. B 85, 085203. 10.1103/physrevb.85.085203

[B50] Hulliger F. Mooser E. (1965b). Semiconductivity in Pyrite, Marcasite and Arsenopyrite Phases. J. Phys. Chem. Sol. 26, 429–433. 10.1016/0022-3697(65)90173-3

[B51] Hulliger F. Mooser E. (1965a). The Bond Description of Semiconductors: Polycompounds. Prog. Solid State. Chem. 2, 330–377. 10.1016/0079-6786(65)90011-7

[B52] Husk D. E. Seehra M. S. (1978). Dielectric Constant of Iron Pyrite (FeS2). Solid State. Commun. 27, 1147–1148. 10.1016/0038-1098(78)91130-4

[B53] Hybertsen M. S. Louie S. G. (1986). Electron Correlation in Semiconductors and Insulators: Band Gaps and Quasiparticle Energies. Phys. Rev. B 34, 5390–5413. 10.1103/physrevb.34.5390 9940372

[B54] Jiang H. Blaha P. (2016). GWwith Linearized Augmented Plane Waves Extended by High-Energy Local Orbitals. Phys. Rev. B 93, 115203. 10.1103/physrevb.93.115203

[B55] Jiang H. (2011). Electronic Band Structure from First-Principles Green's Function Approach: Theory and Implementations. Front. Chem. China 6, 253–268. 10.1007/s11458-011-0261-6

[B56] Jiang H. Gómez-Abal R. I. Li X.-Z. Meisenbichler C. Ambrosch-Draxl C. Scheffler M. (2013). FHI-gap: A GW Code Based on the All-Electron Augmented Plane Wave Method. Comp. Phys. Commun. 184, 348–366. 10.1016/j.cpc.2012.09.018

[B57] Jiang H. (2018). Revisiting the GW Approach to d- and f-electron Oxides. Phys. Rev. B 97, 245132. 10.1103/physrevb.97.245132

[B58] Khalid S. Ahmed E. Khan Y. Nawaz S. Ramzan M. Khalid N. R. (2018). “Iron Pyrite (FeS2): Sustainable Photovoltaic materialMicro and Nanomanufacturing Volume II,”. Editors Jackson M. J. Ahmed W. (Cham: Springer, 281–318. 10.1007/978-3-319-67132-1_11

[B59] Klimes J. Kaltak M. Kresse G. (2014). Predictive GW Calculations Using Plane Waves and Pseudopotentials. Phys. Rev. B 90, 075125.

[B60] Kolb B. Kolpak A. M. (2013). Ultrafast Band-gap Oscillations in Iron Pyrite. Phys. Rev. B 88, 235208. 10.1103/physrevb.88.235208

[B61] Koller D. Blaha P. Tran F. (2013). Hybrid Functionals for Solids with an Optimized Hartree-Fock Mixing Parameter. J. Phys. Condens. Matter 25, 435503. 10.1088/0953-8984/25/43/435503 24107516

[B62] Kotani T. van Schilfgaarde M. (2002). All-electron GW Approximation with the Mixed Basis Expansion Based on the Full-Potential LMTO Method. Solid State. Commun. 121, 461–465. 10.1016/s0038-1098(02)00028-5

[B63] Kou W. W. Seehra M. S. (1978). Optical Absorption in Iron Pyrite (FeS_2_). Phys. Rev. B 18, 7062–7068. 10.1103/physrevb.18.7062

[B64] Krasovskii E. E. (1997). Accuracy and Convergence Properties of the Extended Linear Augmented-Plane-Wave Method. Phys. Rev. B 56, 12866–12873. 10.1103/physrevb.56.12866

[B65] Krasovskii E. E. Yaresko A. N. Antonov V. N. (1994). Theoretical Study of Ultraviolet Photoemission Spectra of noble Metals. J. Electron Spectrosc. Relat. Phenomena 68, 157–166. 10.1016/0368-2048(94)02113-9

[B66] Kresse G. Furthmüller J. (1996). Efficient Iterative Schemes Forab Initiototal-Energy Calculations Using a Plane-Wave Basis Set. Phys. Rev. B 54, 11169–11186. 10.1103/physrevb.54.11169 9984901

[B67] Kronik L. Stein T. Refaely-Abramson S. Baer R. (2012). Excitation Gaps of Finite-Sized Systems from Optimally Tuned Range-Separated Hybrid Functionals. J. Chem. Theor. Comput. 8, 1515–1531. 10.1021/ct2009363 26593646

[B68] Kümmel S. Kronik L. (2008). Orbital-dependent Density Functionals: Theory and Applications. Rev. Mod. Phys. 80, 3–60. 10.1103/revmodphys.80.3

[B69] Laskowski R. Blaha P. (2014). Calculating NMR Chemical Shifts Using the Augmented Plane-Wave Method. Phys. Rev. B 89, 014402. 10.1103/physrevb.89.014402

[B70] Laskowski R. Blaha P. (2012). Calculations of NMR Chemical Shifts with Apw-Based Methods. Phys. Rev. B 85, 035132. 10.1103/physrevb.85.035132

[B71] Lazić P. Armiento R. Herbert F. W. Chakraborty R. Sun R. Chan M. K. (2013). Low Intensity Conduction States in FeS2: Implications for Absorption, Open-Circuit Voltage and Surface Recombination. J. Phys. Condens Matter 25, 465801. 10.1088/0953-8984/25/46/465801 24141033

[B72] Lehner S. W. Newman N. van Schilfgaarde M. Bandyopadhyay S. Savage K. Buseck P. R. (2012). Defect Energy Levels and Electronic Behavior of Ni-, Co-, and As-Doped Synthetic Pyrite (FeS2). J. Appl. Phys. 111, 083717. 10.1063/1.4706558

[B73] Li B. Huang L. Zhong M. Wei Z. Li J. (2015). Electrical and Magnetic Properties of FeS2 and CuFeS2 Nanoplates. RSC Adv. 5, 91103–91107. 10.1039/c5ra16918f

[B74] Li E. K. Johnson K. H. Eastman D. E. Freeouf J. L. (1974). Localized and Bandlike Valence-Electron States in FeS2and NiS2. Phys. Rev. Lett. 32, 470–472. 10.1103/physrevlett.32.470

[B75] Li Y. Chen J. Chen Y. Zhao C. Lee M.-H. Lin T.-H. (2018). DFT+U Study on the Electronic Structures and Optical Properties of Pyrite and Marcasite. Comput. Mater. Sci. 150, 346–352. 10.1016/j.commatsci.2018.04.009

[B76] Limpinsel M. Farhi N. Berry N. Lindemuth J. Perkins C. L. Lin Q. (20141974–1989). An Inversion Layer at the Surface of N-type Iron Pyrite. Energy Environ. Sci. 7. 10.1039/c3ee43169j

[B77] Liu J. Xu A. Meng Y. He Y. Ren P. Guo W.-P. (2019). From Predicting to Correlating the Bonding Properties of Iron Sulfide Phases. Comput. Mater. Sci. 164, 99–107. 10.1016/j.commatsci.2019.04.001

[B78] Liu P. Franchini C. Marsman M. Kresse G. (2020). Assessing Model-dielectric-dependent Hybrid Functionals on the Antiferromagnetic Transition-Metal Monoxides MnO, FeO, CoO, and NiO. J. Phys. Condens. Matter 32, 015502. 10.1088/1361-648x/ab4150 31484169

[B79] Madsen G. K. H. Blaha P. Schwarz K. Sjöstedt E. Nordström L. (2001). Efficient Linearization of the Augmented Plane-Wave Method. Phys. Rev. B 64, 195134. 10.1103/physrevb.64.195134

[B80] Maier T. M. Arbuznikov A. V. Kaupp M. (2019). Local Hybrid Functionals: Theory, Implementation, and Performance of an Emerging New Tool in Quantum Chemistry and beyond. WIREs Comput. Mol. Sci. 9, e1378. 10.1002/wcms.1378

[B81] Mamiya K. Mizokawa T. Fujimori A. Takahashi H. Môri N. Miyadai T. (1997). Photoemission Study of Pyrite-type Transition-Metal Chalcogenides MS2−xSex (M^+^Fe, Co, Ni). Physica B: Condensed Matter 237-238, 390–391. 10.1016/s0921-4526(97)00243-3 9985736

[B82] Marques M. A. L. Vidal J. Oliveira M. J. T. Reining L. Botti S. (2011). Density-based Mixing Parameter for Hybrid Functionals. Phys. Rev. B 83, 035119. 10.1103/physrevb.83.035119

[B83] Marsman M. Paier J. Stroppa A. Kresse G. (2008). Hybrid Functionals Applied to Extended Systems. J. Phys. Condens. Matter 20, 064201. 10.1088/0953-8984/20/6/064201 21693863

[B84] Michalicek G. Betzinger M. Friedrich C. Blügel S. (2013). Elimination of the Linearization Error and Improved Basis-Set Convergence within the Flapw Method. Comp. Phys. Commun. 184, 2670–2679. 10.1016/j.cpc.2013.07.002

[B85] Muscat J. Hung A. Russo S. Yarovsky I. (2002). First-principles Studies of the Structural and Electronic Properties of Pyrite FeS2. Phys. Rev. B 65, 054107. 10.1103/physrevb.65.054107

[B86] Nabok D. Gulans A. Draxl C. (2016). Accurate All-Electron *G* _0_ *W* _0_ Quasiparticle Energies Employing the Full-Potential Augmented Plane-Wave Method. Phys. Rev. B 94, 035118. 10.1103/physrevb.94.035118

[B87] Nesbitt H. W. Uhlig I. Bancroft G. M. Szargan R. (2003). Resonant XPS Study of the Pyrite Valence Band with Implications for Molecular Orbital Contributions. Am. Mineral. 88, 1279–1286. 10.2138/am-2003-8-910

[B88] Ohsawa A. Yamamoto H. Watanabe H. (1974). X-ray Photoelectron Spectra of Valence Electrons in FeS2, CoS2 and NiS2. J. Phys. Soc. Jpn. 37, 568. 10.1143/jpsj.37.568

[B89] Ollonqvist T. Perälä R. Väyrynen J. (1997). Unoccupied Electronic States of the FeS2(100) Surface Studied by Inverse Photoemission. Surf. Sci. 377-379, 201–205. 10.1016/s0039-6028(96)01351-9

[B90] Ouarab N. Boumaour M. (2017). First-principles Calculations of Electronic and Optical Properties of Fe 1−x Zn x S 2 and Zn 1−x Mg x O Alloys. Curr. Appl. Phys. 17, 1169–1180. 10.1016/j.cap.2017.05.008

[B91] Paier J. Marsman M. Hummer K. Kresse G. Gerber I. C. Ángyán J. G. (2006a). Erratum: "Screened Hybrid Density Functionals Applied to Solids" [J. Chem. Phys. 124, 154709 (2006)]. J. Chem. Phys. 125, 249901. J. Chem. Phys. 124, 154709 (2006). 10.1063/1.2403866” 16674253

[B92] Paier J. Marsman M. Hummer K. Kresse G. Gerber I. C. Ángyán J. G. (2006b). Screened Hybrid Density Functionals Applied to Solids. J. Chem. Phys. 124, 154709. 10.1063/1.2187006 16674253

[B93] Perdew J. P. Burke K. Ernzerhof M. (1996a). Generalized Gradient Approximation Made Simple. Phys. Rev. Lett. 77, 3865–3868. 10.1103/physrevlett.77.3865 10062328

[B94] Perdew J. P. Ernzerhof M. Burke K. (1996b). Rationale for Mixing Exact Exchange with Density Functional Approximations. J. Chem. Phys. 105, 9982–9985. 10.1063/1.472933

[B95] Perdew J. P. Parr R. G. Levy M. Balduz J. L. (1982). Density-functional Theory for Fractional Particle Number: Derivative Discontinuities of the Energy. Phys. Rev. Lett. 49, 1691–1694. 10.1103/physrevlett.49.1691

[B96] Perdew J. P. Schmidt K. (2001). Jacob’s Ladder of Density Functional Approximations for the Exchange-Correlation Energy. AIP Conf. Proc. 577, 1. 10.1063/1.1390175

[B97] Perdew J. P. Yang W. Burke K. Yang Z. Gross E. K. U. Scheffler M. (2017). Understanding Band Gaps of Solids in Generalized Kohn-Sham Theory. Proc. Natl. Acad. Sci. USA 114, 2801–2806. 10.1073/pnas.1621352114 28265085 PMC5358356

[B98] Pickett W. E. Krakauer H. Allen P. B. (1988). Smooth Fourier Interpolation of Periodic Functions. Phys. Rev. B 38, 2721–2726. 10.1103/physrevb.38.2721 9946584

[B99] Rahman M. Boschloo G. Hagfeldt A. Edvinsson T. (2020). On the Mechanistic Understanding of Photovoltage Loss in Iron Pyrite Solar Cells. Adv. Mater. 32, 1905653. 10.1002/adma.201905653 32424936

[B100] Ren X. Merz F. Jiang H. Yao Y. Rampp M. Lederer H. (2021). All-electron Periodic *G* _0_ *W* _0_ Implementation with Numerical Atomic Orbital Basis Functions: Algorithm and Benchmarks. Phys. Rev. Mater. 5, 013807. 10.1103/physrevmaterials.5.013807

[B101] Ren X. Rinke P. Blum V. Wieferink J. Tkatchenko A. Sanfilippo A. (2012). Resolution-of-identity Approach to Hartree-Fock, Hybrid Density Functionals, RPA, MP2 andGWwith Numeric Atom-Centered Orbital Basis Functions. New J. Phys. 14, 053020. 10.1088/1367-2630/14/5/053020

[B102] Rojas H. N. Godby R. W. Needs R. J. (1995). Space-Time Method forAb InitioCalculations of Self-Energies and Dielectric Response Functions of Solids. Phys. Rev. Lett. 74, 1827–1830. 10.1103/physrevlett.74.1827 10057767

[B103] Sánchez C. Flores E. Barawi M. Clamagirand J. M. Ares J. R. Ferrer I. J. (2016). Marcasite Revisited: Optical Absorption gap at Room Temperature. Solid State. Commun. 230, 20–24. 10.1016/j.ssc.2016.01.004

[B104] Schena T. Bihlmayer G. Blügel S. (2013). First-Principles Studies of FeS_2_ Using Many-Body Perturbation Theory in the G0W0 Approximation. Phys. Rev. B 88, 235203. 10.1103/physrevb.88.235203

[B105] Schlegel A. Wachter P. (1976). Optical Properties, Phonons and Electronic Structure of Iron Pyrite (FeS2). J. Phys. C: Solid State. Phys. 9, 3363–3369. 10.1088/0022-3719/9/17/027

[B106] Seidl A. Görling A. Vogl P. Majewski J. A. Levy M. (1996). Generalized Kohn-Sham Schemes and the Band-gap Problem. Phys. Rev. B 53, 3764–3774. 10.1103/physrevb.53.3764 9983927

[B107] Shen T. Zhang X.-W. Shang H. Zhang M.-Y. Wang X. Wang E.-G. (2020). Influence of High-Energy Local Orbitals and Electron-Phonon Interactions on the Band Gaps and Optical Absorption Spectra of Hexagonal boron Nitride. Phys. Rev. B 102, 045117. 10.1103/physrevb.102.045117

[B108] Shimazaki T. Asai Y. (2008). Band Structure Calculations Based on Screened Fock Exchange Method. Chem. Phys. Lett. 466, 91–94. 10.1016/j.cplett.2008.10.012 20550388

[B109] Shishkin M. Kresse G. (2007). Self-consistentGWcalculations for Semiconductors and Insulators. Phys. Rev. B 75, 235102. 10.1103/physrevb.75.235102

[B110] Shukla S. Xing G. Ge H. Prabhakar R. R. Mathew S. Su Z. (2016). Origin of Photocarrier Losses in Iron Pyrite (FeS2) Nanocubes. ACS Nano 10, 4431–4440. 10.1021/acsnano.6b00065 26962638

[B111] Singh D. (1991). Ground-state Properties of Lanthanum: Treatment of Extended-Core States. Phys. Rev. B 43, 6388–6392. 10.1103/physrevb.43.6388 9998076

[B112] Singh D. J. Nordström L. (2006). Planewaves, Pseudopotentials and the LAPW Method. 2nd ed. edn. New York: Springer.

[B113] Skone J. H. Govoni M. Galli G. (2014). Self-consistent Hybrid Functional for Condensed Systems. Phys. Rev. B 89, 195112. 10.1103/physrevb.89.195112

[B114] Spagnoli D. Refson K. Wright K. Gale J. D. (2010). Density Functional Theory Study of the Relative Stability of the Iron Disulfide Polymorphs Pyrite and Marcasite. Phys. Rev. B 81, 094106. 10.1103/physrevb.81.094106

[B115] Stankovski M. Antonius G. Waroquiers D. Miglio A. Dixit H. Sankaran K. (2011). G0W0band gap of ZnO: Effects of Plasmon-Pole Models. Phys. Rev. B 84, 241201. (R). 10.1103/physrevb.84.241201

[B116] Sun R. Chan M. K. Y. Ceder G. (2011). First-principles Electronic Structure and Relative Stability of Pyrite and Marcasite: Implications for Photovoltaic Performance. Phys. Rev. B 83, 235311. 10.1103/physrevb.83.235311

[B117] Tian A. Xu Q. Shi X. Yang H. Xue X. You J. (2015). Pyrite Nanotube Array Films as an Efficient Photocatalyst for Degradation of Methylene Blue and Phenol. RSC Adv. 5, 62724–62731. 10.1039/c5ra07434g

[B118] van der Heide H. Hemmel R. van Bruggen C. F. Haas C. (1980). X-ray Photoelectron Spectra of 3d Transition Metal Pyrites. J. Solid State. Chem. 33, 17–25. 10.1016/0022-4596(80)90543-5

[B119] van Setten M. J. Giantomassi M. Bousquet E. Verstraete M. J. Hamann D. R. Gonze X. (2018). The PseudoDojo: Training and Grading a 85 Element Optimized Norm-Conserving Pseudopotential Table. Comp. Phys. Commun. 226, 39–54. 10.1016/j.cpc.2018.01.012

[B120] van Setten M. J. Giantomassi M. Gonze X. Rignanese G.-M. Hautier G. (2017). Automation Methodologies and Large-Scale Validation for GW : Towards High-Throughput GW Calculations. Phys. Rev. B 96, 155207. 10.1103/physrevb.96.155207

[B121] Wadia C. Alivisatos A. P. Kammen D. M. (2009). Materials Availability Expands the Opportunity for Large-Scale Photovoltaics Deployment. Environ. Sci. Technol. 43, 2072–2077. 10.1021/es8019534 19368216

[B122] Walter J. Zhang X. Voigt B. Hool R. Manno M. Mork F. (2017). Surface Conduction in N-type Pyrite FeS2 Single Crystals. Phys. Rev. Mater. 1, 065403. 10.1103/physrevmaterials.1.065403

[B123] Wang D.-Y. Jiang Y.-T. Lin C.-C. Li S.-S. Wang Y.-T. Chen C.-C. (2012). Solution-processable Pyrite FeS2 Nanocrystals for the Fabrication of Heterojunction Photodiodes with Visible to NIR Photodetection. Adv. Mater. 24, 3415–3420. 10.1002/adma.201200753 22674518

[B124] Wilson J. A. (1972). Systematics of the Breakdown of Mott Insulation in Binary Transition Metal Compounds. Adv. Phys. 21, 143–198. 10.1080/00018737200101278

[B125] Wu L. Dzade N. Y. Gao L. Scanlon D. O. Öztürk Z. Hollingsworth N. (2016). Enhanced Photoresponse of FeS2Films: The Role of Marcasite-Pyrite Phase Junctions. Adv. Mater. 28, 9602–9607. 10.1002/adma.201602222 27628579

[B126] Zhang M.-Y. Cui Z.-H. Jiang H. (2018). Relative Stability of FeS_2_ Polymorphs with the Random Phase Approximation Approach. J. Mater. Chem. A. 6, 6606–6616. 10.1039/c8ta00759d

[B127] Zhang M.-Y. Jiang H. (2019). Electronic Band Structure of Cuprous and Silver Halides: An All-Electron *GW* Study. Phys. Rev. B 100, 205123. 10.1103/physrevb.100.205123

[B128] Zhang M. Y. Cui Z. H. Wang Y. C. Jiang H. (2020). Hybrid Functionals with System‐dependent Parameters: Conceptual Foundations and Methodological Developments. WIREs Comput. Mol. Scimol. Sci. 10, 1476. 10.1002/wcms.1476

[B129] Zuñiga-Puelles E. Cardoso-Gil R. Bobnar M. Veremchuk I. Himcinschi C. Hennig C. (2019). Structural Stability and Thermoelectric Performance of High Quality Synthetic and Natural Pyrites (FeS2). Dalton Trans. 48, 10703–10713. 10.1039/c9dt01902b 31243411

